# Proteomic Characterization of Bacteriophage Peptides from the Mastitis Producer *Staphylococcus aureus* by LC-ESI-MS/MS and the Bacteriophage Phylogenomic Analysis

**DOI:** 10.3390/foods10040799

**Published:** 2021-04-08

**Authors:** Ana G. Abril, Mónica Carrera, Karola Böhme, Jorge Barros-Velázquez, Benito Cañas, José-Luis R. Rama, Tomás G. Villa, Pilar Calo-Mata

**Affiliations:** 1 Department of Microbiology and Parasitology, Faculty of Pharmacy, University of Santiago de Compostela, 15898 Santiago de Compostela, Spain; anagonzalezabril@hotmail.com (A.G.A.); joserodrama@gmail.com (J.-L.R.R.); tomas.gonzalez@usc.es (T.G.V.); 2 Department of Food Technology, Spanish National Research Council, Marine Research Institute, 36208 Vigo, Spain; 3 Agroalimentary Technological Center of Lugo, 27002 Lugo, Spain; KarolaBoehme@gmx.de; 4 Department of Analytical Chemistry, Nutrition and Food Science, School of Veterinary Sciences, University of Santiago de Compostela, 27002 Lugo, Spain; jorge.barros@usc.es; 5 Department of Analytical Chemistry, Complutense University of Madrid, 28040 Madrid, Spain; bcanas@quim.ucm.es

**Keywords:** pathogen detection, LC-ESI-MS/MS, proteomics, mass spectrometry, phage peptide biomarker

## Abstract

The present work describes LC-ESI-MS/MS MS (liquid chromatography-electrospray ionization-tandem mass spectrometry) analyses of tryptic digestion peptides from phages that infect mastitis-causing *Staphylococcus aureus* isolated from dairy products. A total of 1933 nonredundant peptides belonging to 1282 proteins were identified and analyzed. Among them, 79 staphylococcal peptides from phages were confirmed. These peptides belong to proteins such as phage repressors, structural phage proteins, uncharacterized phage proteins and complement inhibitors. Moreover, eighteen of the phage origin peptides found were specific to *S. aureus* strains. These diagnostic peptides could be useful for the identification and characterization of *S. aureus* strains that cause mastitis. Furthermore, a study of bacteriophage phylogeny and the relationship among the identified phage peptides and the bacteria they infect was also performed. The results show the specific peptides that are present in closely related phages and the existing links between bacteriophage phylogeny and the respective *Staphylococcus* spp. infected.

## 1. Introduction

The vast majority of mastitis cases are due to an intramammary infection caused by a microorganism belonging to either the *Staphylococcus* or *Streptococcus* genus [1,2]. *Staphylococcus aureus* is considered one of the major foodborne pathogens that can cause serious food intoxication in humans due to the production of endotoxins; this pathogen remains a major issue in the dairy industry due to its persistence in cows, its pathogenicity, its contagiousness and its ease of colonization of the skin and mucosal epithelia [3,4,5].

It is well-known that *S. aureus* bacteriophages encode genes for staphylococcal virulence factors, such as Panton-Valentine leucocidin, staphylokinase, enterotoxins, chemotaxis-inhibitory proteins or exfoliative toxins [6]. These phages are usually integrated into bacterial chromosomes as prophages, wherein they encode new properties in the host, or vice versa, as transcriptions may hardly be affected by gene disruptions [7]. Phage-encoded recombinases, rather than the host recombinase, RecA, are involved in bacterial genome excisions and integrations [8,9]. These integrations may occur at specific bacterial genome sites that are identical to those present in the DNA of the phage, or, as in the case of phage Mu (as long as the given gene is not expressed), some phages can integrate randomly within the bacterial genome. In addition, bacteriophage and staphylococcal species interactions may substantially alter the variability of the bacterial population [10,11].

All known *S. aureus* phages are composed of an icosahedral capsid filled with double-stranded DNA and a thin, filamentous tail, and they belong to the order *Caudovirales* (tailed phages) [12,13]. Some *Podoviridae* family phages, such as the *Staphylococcus* viruses S13′ and S24-1, have been reported, characterized and used in phage therapy against *S. aureus* infections [14]. There are some well-known *Siphoviridae* phages of *S. aureus*, such as the prophage φSaBov, which is integrated into a bovine mastitis-causing *S. aureus* strain [15].

The interaction between bacteria and bacteriophages leads to an exchange of genetic information, which enables bacteria to rapidly adapt to challenging environmental conditions and to be highly dynamic [11,16]. As closely related phages normally occupy the same genome location in different bacteria, a specific site in different bacterial strains can be occupied by completely different phages or can be empty.

Conventional culture-based methods have been used for the detection of pathogenic bacteria [17,18] and their phages [19,20]; however, at this point, these procedures are time-consuming and laborious. For this reason, new, rapid molecular microbial diagnostic methods based on genomics and proteomics tools have been developed to achieve faster and more efficient bacterial and bacteriophage identification [1,21,22,23,24]. Specifically, phage typing is a classic technique for such purposes [25]. Moreover, biosensors based on phage nucleic acids, receptor-binding proteins (RBPs), antibodies and phage display peptides (PDPs) have been used for pathogen detection [26,27,28,29,30].

Mass spectrometry techniques, such as MALDI-TOF MS (matrix-assisted laser desorption/ionization time-of-flight mass spectrometry) and LC-ESI-MS/MS (liquid chromatography-electrospray ionization-tandem mass spectrometry), have been used for the analysis and detection of specific diagnostic peptides in pathogenic bacterial strains [31,32]. In addition, LC-ESI-MS/MS methods have been employed for the identification and detection of bacteriophages [19]. In the case of bacteriophage detection and identification by a mass spectrometry analysis, the required production of viruses may be time-consuming. The detection of prophages based on protein biomarkers can be an alternative to genomic detection, and in this sense, proteomic techniques can be cheaper and faster and can ascertain different bacteriophage species by using a single analysis [33]. Based on the specificity of many bacteriophages with their hosts, bacteriophages are considered signal amplifiers; therefore, the detection of peptides from phages is suitable for pathogen identification. For example, Serafim et al. 2017 [33] identified bacteriophage lambda by a LC-ESI-MS/MS analysis. Moreover, the identification of peptides by means of LC-ESI-MS/MS from bacteriophage-infected *Streptococcus* has been performed, which revealed new information on phage phylogenomics and their interactions with the bacteria they infect [19]. However, no study has been published on *S. aureus* phage detection and identification by LC-ESI-MS/MS or on *S. aureus* phage characterization without a previous phage purification step. Viral genomic detection and phage display are time-consuming methods. Here, we describe an easy, fast and accurate method for the detection of bacteriophages without the need for the pretreatment of bacterial lysis for bacteriophage replication. This method led to the identification of putative temperate and virulent phages present in the analyzed strains.

A previously published work performed by our laboratory [3] studied the global proteome of several strains of *S. aureus* by shotgun proteomics. Important virulence protein factors and functional pathways were characterized by a protein network analysis. In this work, and for the first time, we aimed to use proteomics to characterize phage contents in different *S. aureus* strains to identify the relevant phage-specific peptides of several *S. aureus* strains and to identify both phages and bacterial strains by LC-ESI-MS/MS.

## 2. Materials and Methods

### 2.1. Bacteria

In this study, a total of 20 different *S. aureus* strains obtained from different sources were analyzed (Table S1 in Supplemental Data 2). These strains were previously characterized by MALDI-TOF mass spectrometry [1] after being obtained from the Institute of Science of Food Production of the National Research Council of Italy (Italy) and from the Spanish Type Culture Collection (Spain). The majority of the strains are from food origins, except for strain U17, which is a human clinical strain. Strains ATCC (American Type Culture Collection) 9144 and ATCC 29213 are classified as *S. aureus* subsp. *aureus*, while strain ATCC 35845 is categorized as *S. aureus* subsp. *anaerobius*. In previous works, the species identification of *S. aureus* and the presence of enterotoxins were evaluated by multiplex polymerase chain reactions (multiplex PCRs) [3,34,35]. The strains were reactivated in a brain–heart infusion medium (BHI, Oxoid Ltd., Hampshire, UK) and incubated at 31 °C for 24 h. Bacterial cultures were then grown on plate count agar (PCA, Oxoid) at 31 °C for 24 h [1,3,36]. Tubes of broth were inoculated under aerobic conditions.

### 2.2. Protein Extraction and Peptide Sample Preparation

Protein extraction was prepared as described previously [37]. All analyses were performed in triplicate. Protein extracts were subjected to in-solution tryptic digestion [38].

### 2.3. Shotgun LC-MS/MS Analysis

Peptide digests were acidified with formic acid (FA), cleaned on a C18 MicroSpin™ column (The Nest Group, South-borough, MA, USA) and analyzed by LC-ESI-MS/MS using a Proxeon EASY-nLC II Nanoflow system (Thermo Fisher Scientific, San Jose, CA, USA) coupled to an LTQ-Orbitrap XL mass spectrometer (Thermo Fisher Scientific, San Jose, CA, USA) [3]. Peptide separation (2 μg) was performed on a reverse-phase (RP) column (EASY-Spray column, 50 cm × 75 μm ID, PepMap C18, 2-μm particles, 100-Å pore size, Thermo Fisher Scientific, San Jose, CA, USA) with a 10-mm precolumn (Accucore XL C18, Thermo Fisher Scientific, San Jose, CA, USA) using a linear 120-min gradient from 5% to 35% solvent B (solvent A: 98% water, 2% ACN (Acetonitrile) and 0.1% FA and solvent B: 98% ACN, 2% water and 0.1% FA) at a flow rate of 300 nL/min. For ionization, a spray voltage of 1.95 kV and a capillary temperature of 230 °C were used. Peptides were analyzed in the positive mode from 400 to 1600 amu (1 μscan), which was followed by 10 data-dependent collision-induced dissociation (CID) MS/MS scans (1 μscan) using an isolation width of 3 amu and a normalized collision energy of 35%. Fragmented masses were set in dynamic exclusion for 30 s after the second fragmentation event, and unassigned charged ions were excluded from the MS/MS analysis.

### 2.4. LC-MS/MS Mass Spectrometry Data Processing

LC-ESI-MS/MS spectra were searched using SEQUEST-HT (Proteome Discoverer 2.4, Thermo Fisher Scientific, San Jose, CA, USA) against the *S. aureus* UniProt/TrEMBL database (208,158 protein sequence entries in July 2020). The following parameters were used: semi-tryptic cleavage with up to two missed cleavage sites and tolerance windows set at 10 ppm for the precursor ions and 0.06 Da for the MS/MS fragment ions. These additional identified semi-tryptic peptides increased the sequence coverage and confidence in protein assignments. The variable modifications that were allowed were as follows: (M*) methionine oxidation (+15.99 Da), (C*) carbamidomethylation of Cys (+57.02 Da) and acetylation of the N-terminus of the protein (+42.0106 Da). To validate the peptide assignments, the results were subjected to a statistical analysis with the Percolator algorithm [39]. The false discovery rate (FDR) was kept below 1%. The mass spectrometric data were deposited into the public database PRIDE (Proteomics Identification Database), with the dataset identifier PXD023530.

### 2.5. Selection of Potential Peptide Biomarkers

For each peptide identified by LC-ESI-MS/MS, we used the BLASTp program to determine the homologies and exclusiveness with protein sequences registered in the NCBI (National Center for Biotechnology Information) database [40]. For the BLASTp search, the *Staphylococcus* taxon was included and excluded with the aim of finding the peptides that belonged to the *Staphylococcus* phages, *Staphylococcus* spp. and only to *S. aureus*.

### 2.6. Phage Genome Comparison and Relatedness

Genomes of all studied *Staphylococcus* spp. phages were downloaded from the GenBank database, analyzed and compared using the Web server VICTOR (Virus Classification and Tree Building Online Resource, http://ggdc.dsmz.de/victor.php, accessed on 27 November 2020) for the calculation of the intergenomic distances and the construction of the phylogenomic tree [41].

## 3. Results

### 3.1. S. aureus Proteome Repository

Protein mixtures from each of the 20 different *S. aureus* strains (Table S1 in Supplemental Data 2) were digested with trypsin and analyzed by LC-ESI-MS/MS.

**Figure 1:**
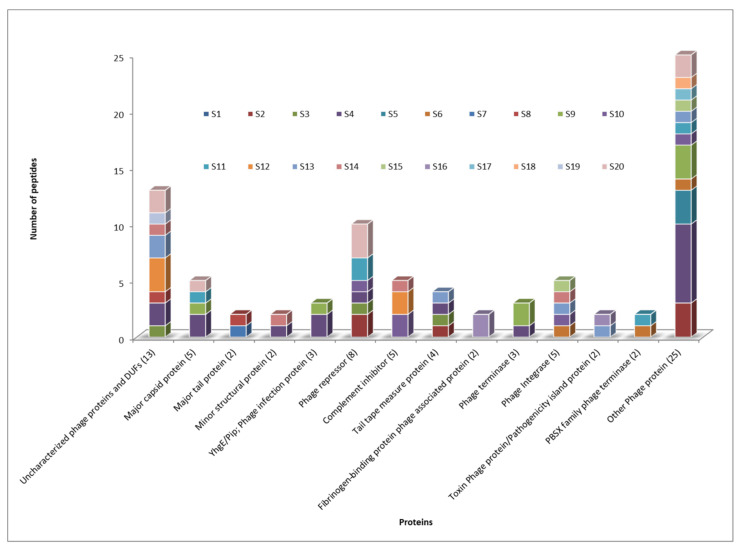
Comparative representation of different types of phage proteins identified in this study for the different strains (represented by different colors). The number of each type of protein is shown in parentheses.

A total of 1933 nonredundant peptides corresponding to 1282 nonredundant annotated proteins were identified for all *S. aureus* strains (see the Excel dataset in Supplemental Data 1). Among them, 79 phage peptides were identified. These peptides belong to proteins such as phage repressors, structural phage proteins, uncharacterized phage proteins and complement inhibitors. Figure 1 shows a comparative representation of the different types of phage proteins identified in this study. These phage peptides were selected and analyzed using the BLASTp algorithm. For the BLASTp search, *Staphylococcus* was included and excluded with the aim of finding peptides belonging to *Staphylococcus* bacteriophages.

The obtained staphylococcal phage-specific peptides shared homology with the *Staphylococcus* phages and *Staphylococcus* spp. in the NCBI database. Among them, all shared homology with *S. aureus*; however, eighteen peptides were specific to *S. aureus* (IRLPYYDVK, LYVGVFNPEATK, SIINGKLDSQWTVPNEHK, M*NDSNQGLQANPQYTIHYLSQEITR, PCPALM*NKRNSIATIHR, SQDSNLTPELSTKAPK, ESINANTYINQNLEK, VAVLSTPLVTSFESK, KDGEILFDAIDIYLRNK, MPVYKDGNTGKWYFSI, KTTSEALKEVLSDT, EPKPVDATGADDPLKPDDRM*ITNFHANLVDQKVSY, MSHNALTTGIGIGAGAG, VQHPGKLVNKVM*SGLNINFGGGANATAK, QM*MEGLSGVMDLAAVSGEDLGAVSDIVTDGLTAFGLKAKDSG, KSNVEAFSNAVK, GMVASMQMQVVQVNVLTM*ELAQQNAMLTQQLTELK and DIITVYC*PENGTATDEY). Figure S1 shows the MS/MS spectra for these *S. aureus*-specific peptide biomarkers. Table 1 summarizes the list of 79 specific staphylococcal bacteriophage peptides, bacterial peptides with putative phage origins and bacteria and phages with 100% homology with respect to the NCBI protein database.

All staphylococcal phage peptides with 100% homology were found to belong to the *Siphoviridae* family: 52 staphylococcal phages belong to the *Phietavirus* genus, 37 belong to the *Biseptimavirus* genus, 30 are *Triavirus*, two are phieta-like viruses and one is a SPbeta-like virus, and the others are nonclassified *Siphoviridae* viruses (Table S2 in Supplemental Data 2). *Siphoviridae* genomes are usually organized into functional modules, such as lysogeny, DNA replication, packaging, morphogenesis and lysis modules [6,42].

### 3.2. Phage Peptides Determined from the Analyzed S. aureus Strains

For strains S2 and S3, six and three phage peptides were determined, respectively. For strain S4, seventeen phage peptides were determined, and three phage peptides were determined for strain S5. For strains S6 and S7, three and one phage peptides were determined, respectively. Moreover, for strains S8 and S9, two phage peptides and seven phage peptides were determined. For strains S10 and S11, five and three phage peptides were determined, respectively. For strains S12 and S13, five phage peptides and six phage peptides were determined, respectively. For strains S14 and S15, four and two phage peptides were determined, respectively. For strain S16, three phage peptides were determined, and one phage peptide was determined for strain S17. For strains S18 and S19, one phage peptide each was determined. Finally, for strain S20, seven phage peptides were determined.

A large number of phage peptides from structural proteins were identified (Table 1). Peptides from proteins such as the major capsid protein, major tail protein, minor structural protein, phage head morphogenesis protein, tail tape measure protein and phage tail fiber protein were determined. Moreover, different phage peptides from the major capsid protein and tail protein were determined (Table 1). Identifying these phage peptides is reasonable, as the major capsid protein and major tail protein are the most abundant proteins in mature virions [6].

There are a large number of uncharacterized protein sequences in databases, and more than 20% of all protein domains are annotated as “domains of unknown function” (DUFs). Several uncharacterized phage proteins and DUFs from *Staphylococcus* bacteriophages were identified for the analyzed strains (Table 1) [43,44].

Different peptides from repressor-type Cro/CI were determined. For strains S11 and S20 (both potential enterotoxin C producers), the same phage peptides of repressor-type Cro/CI were identified (Table 1). CI and Cro are encoded in the lysogeny module of lambdoid bacteriophages, particularly λ bacteriophages. Together, CII and CIII (that are formed through the anti-terminator role of protein N) act as an inducer that favors the first expression of the *cI* gene from the appropriate promoter; if the CI repressor predominates, the phage remains in the lysogenic state, but if the Cro predominates, the phage transitions into the lytic cycle, helped by the late Q regulator. The xenobiotic XRE regulator is extended in bacteria and has similarity to the Croλ repressor, exhibiting a helix-turn-helix (HTH) conformation [45]. Peptides of the CI/Cro-repressor types are usually named XRE family proteins in the NCBI database for bacteria.

Three phage peptides of the complement inhibitor were identified (Table 1). Staphylococcal complement inhibitors are involved in the evasion of human phagocytosis by blocking C3 convertases, and a study reported that complement inhibitor genes were also found in *staphylococcal* phages [46]. Another autolysin was determined in the present results, an N-acetylmuramoyl-L-alanine amidase that plays a role in bacterial adherence to eukaryotic cells [19]. The phage protein NrdI, which is a type of ribonucleotide reductase (RNR), was also identified. Several peptides of transposases, integrases and terminases were identified along with a DNA primase phage associated protein and a DNA phage binding protein. Moreover, peptides of other proteins, such as GNAT family N-acetyltransferase, holin, peptidase, methylase, anti-repressor protein (Ant), phage-resistant protein, phage-encoded lipoprotein, phage infection protein, phage portal protein, toxin phage proteins associated with pathogenicity islands and a protein involved in fibrinogen-binding proteins, were identified. A PBSX family phage terminase peptide was determined, and this protein is involved in double-stranded DNA binding, DNA packaging and endonuclease and ATPase activities [47].

As shown in Table 1, the vast majority of phage-specific peptides are not specific to *S. aureus* and can be found in other species of *Staphylococcus*. As an exception, the same peptides, such as peptide LLHALPTGNDSGGDKLLPK from a major capsid protein, were also found in *Streptococcus pneumoniae*, and peptide AYINITGLGFAK from a major tail protein was also found in *Pararheinheimera mesophila*; whether these examples represent direct recombinations between bacteria belonging to different families or whether phage-mediated recombination occurs remains to be elucidated. Furthermore, as mentioned before, eighteen identified peptides were very specific for *S. aureus* based on the NCBI database (see Figure S1).

### 3.3. Staphylococcus spp. Phage Genome Comparisons and Their Relatedness

**Figure 2:**
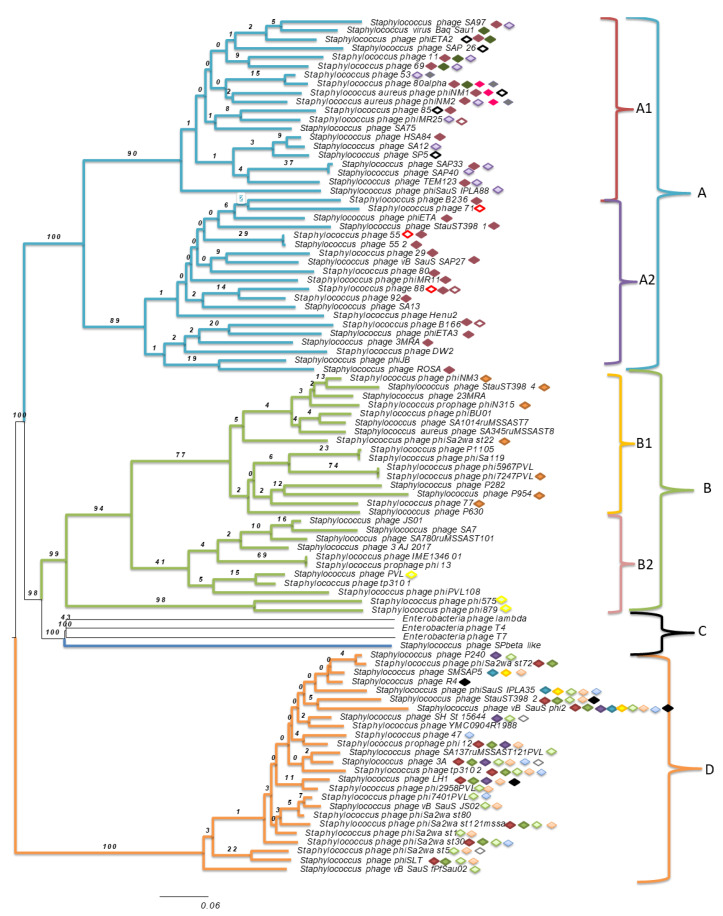
Phylogenomic tree generated by the Virus Classification and Tree Building Online Resource (VICTOR) using the complete genomic sequences of the determined *Staphylococcus* spp. phages. The access numbers of the determined phage genomes are shown in Table S2 in Supplemental Data 2. Genomes of the *lambda* (NC_001416.1), *T4* (NC_000866.4) and *T7* (NC_001604.1) phages were added for comparison purposes. The VICTOR phylogenetic tree construction was based on an intergenic distance analysis with the GBDP tool (Genome BLAST Distance Phylogeny). The significance of each branch is indicated by a pseudo-bootstrap value calculated as a percentage for 1000 subsets. Bar, 20 nt (nucleotides) substitutions per 100 nt. Clusters are represented by different colors: light blue, cluster A, red, cluster A.1, purple, cluster A.2, light green, cluster B, yellow, cluster B.1, pink, cluster B.2, black, cluster C and orange, cluster D. Specific cluster peptides are represented by different color forms: 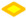, yellow-filled diamond IQQLADYFNVPK (cluster A-specific), 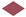, brown-filled diamond HAGYVRC*KLF (cluster A-specific), 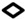, black-outlined diamond IYDRNSDTLDGLPVVNLK (cluster A.1-specific), 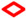, red=outlined diamond AVAELLKEINR (cluster A.2-specific), 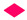, pink-filled diamond KSNVEAFSNAVK (cluster A.1), 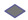, gray-filled diamond QKNVLNYANEQLDEQNKV (cluster A.1), 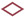, brown-outlined diamond MPVYKDGNTGKWYFSI (cluster A-specific), 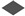, dark gray-filled diamond KLYIIEEYVKQGM (cluster A.1-specific), 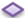, purple-outlined diamond EVPNEPDYIVIDVC*EDYSASK (cluster A.1-specific), 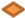, orange-filled diamond AYINITGLGFAK (cluster B.1-specific), 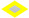, yellow-outlined diamond TSIELITGFTK (cluster B.2-specific), 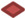, red-filled diamond VSYTLDDDDFITDVETAK (cluster D-specific), 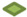, green-filled diamond LLHALPTGNDSGGDKLLPK (cluster D-specific), 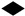, black-filled diamond RVSYTLDDDDFITDVETAKELKL (cluster D-specific), 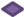, purple-filled diamond LYVGVFNPEATK (cluster D-specific, 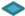, blue-filled diamond ELAEAIGVSQPTVSNWIQQTK (cluster D-specific); 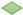, light green-filled diamond VLEMIFLGEDPK (cluster D-specific), 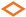, orange-outlined diamond KAMIKASPK (cluster D-specific) and 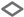, gray-outlined diamond GMPTGTNVYAVKGGIADK (cluster D-specific).

A phylogenomic tree of *Staphylococcus* spp. phages from the NCBI database (accession numbers in Table S2 in Supplemental Data 2) with 100% similarity to those found in this study was built (Figure 2). The phages identified in this study were classified in the order *Caudovirales* and the family *Siphoviridae*. Many of these bacteriophages were classified into the genera *Phietavirus*, *Biseptimavirus, Triavirus* phieta-like virus, SPbeta-like virus and unclassified genera. Genomes of well-known phages of the families *Siphoviridae*, *Myoviridae* and *Podoviridae*, such as phage Lambda, T4 and T7, respectively, were added for comparison purposes. The genome analysis showed three well-defined clusters that mainly divided the phylogenomic tree into different phage genera (*Phietavirus*, *Biseptimavirus* and *Triavirus*). Two principal branches separated Clusters A, B and C from D. Cluster A was formed by *Staphylococcus Phietavirus,* two phieta-like viruses and two unclassified *Staphylococcus* phages. Cluster B was formed by *Staphylococcus* phages classified as *Biseptimavirus* and by one unclassified *Staphylococcus* phage. Cluster C was formed by enterobacterial bacteriophages and one SPbeta-like virus. Finally, cluster D was formed by *Triavirus Staphylococcus* phages and two unclassified *Staphylococcus* phages. To the best of our knowledge, this is the first time that phages from mastitis-causing staphylococci were grouped in a phylogenomic tree.

Specific peptides were found in related *Staphylococcus* spp. phages (Table 2) located closely in the phylogenomic tree (Figure 2). Peptides HAGYVRC*KLF and MPVYKDGNTGKWYFSI were found in phages of cluster A. Furthermore, peptides IYDRNSDTLDGLPVVNLK, QKNVLNYANEQLDEQNKV, EVPNEPDYIVIDVC*EDYSASK, KSNVEAFSNAVK and KLYIIEEYVKQGM were found in *Staphylococcus* phages of the A.1 subbranch in cluster A. Additionally, peptide AVAELLKEINR was found in phages of the A.2 branch. The peptide AYINITGLGFAK was found in phages of cluster B.1, and TSIELITGFTK was found in phages of cluster B.2. Peptides VSYTLDDDDFITDVETAK and LLHALPTGNDSGGDKLLPK, which belong to the phage major capsid protein, were found in the same 14 *Staphylococcus* phages of cluster D. Peptides ELAEAIGVSQPTVSNWIQQTK and IQQLADYFNVPK, which belong to the phage-repressor Cro/CI family of proteins, were found in the same bacteriophages of cluster D. Moreover, peptides LYVGVFNPEATK, RVSYTLDDDDFITDVETAKELKL LYVGVFNPEATK, VLEMIFLGEDPK, KAMIKASPK, EFRNKLNELGADK and GMPTGTNVYAVKGGIADK were also found in phages of cluster D. Peptides IHDKELDDPSEEESKLTQEEENSI, IIINHDEIDLL, KDRYSSVSY and AEEAGVTVKQL are specific to *Staphylococcus* phage SPbeta-like.

In addition, a correlation relating bacterial species for each cluster with all peptides found in the bacteriophages with 100% similarity was found. The results showed that clustered phages were related to specific species of *Staphylococcus*. All studied phages were found to be related to *S. aureus*; however, most of them were also found to be related to additional *Staphylococcus* species. *S. argenteus* was found to be related in all clusters of the phylogenomic tree. Cluster A phage peptides were found to be mainly related to *S. simiae*. However, different *Staphylococcus* species (*S. xylosus*, *S. muscae*, *S. haemolyticus*, *S. simiae*, *S. sciuri*, *S. pseudintermedius, S. devriesei*, *S. warneri* and *S. capitis*) were found to be related to phages of cluster D.

### 3.4. Identification of Peptides of Virulence Factors

In this work, 405 peptides from *S. aureus* were determined to be related to virulence factors (Excel dataset Supplemental Data). Among these peptides, proteins such as staphopain, beta-lactamase, elastin-binding protein peptides and a multidrug ATP-binding cassette (ABC) transporter were identified.

## 4. Discussion

LC-MS/MS-based methods for bacteriophage identification offer several advantages compared with other approaches, since bacteriophages can be directly identified with this method without using genomic tools, which provides a new strategy for drawing the appropriate conclusions. In addition, the method proposed here may be applied for further analyses without the requirement of growing bacteria, since the samples can be collected directly from foodstuffs. The study of noninduced prophages provides a fast analysis and can detect specific temperate phage proteins produced by *S. aureus* while integrated in the bacterial genome or by phages that are infecting the bacteria. Both cases provide the identification of specific *S. aureus* species or strains—in this case, an *S. aureus* mastitis producer. In the proteomic repository of the 20 different *S. aureus* strains analyzed, 79 peptides from staphylococcal bacteriophages were identified. Among them, eighteen of these phage peptides were *S. aureus*-specific. As bacteriophages are host-specific, these putative diagnostic peptides could be good diagnostic biomarkers for the detection and characterization of *S. aureus* and *S. aureus* phages.

The results show that a given specific peptide is present in closely related phages (Table 2). These bacteriophage peptides can be used as specific markers to establish *S. aureus* bacteriophage relationships (Figure 2). Additionally, phages that show the same peptides and are specific to *Staphylococcus* spp. are located close to one another in the phylogenomic tree, suggesting that a link does exist between phage phylogeny and bacteriophages that can infect the same bacterial species.

The study shown here exemplifies how phylogenomic trees based on the genome analysis provide useful information, and the study corroborates previous investigations, which suggested that viral genomic or subgenomic region analyses provide the best tool for reconstructing viral evolutionary histories [48]. Nevertheless, the lack of knowledge of the phage genomic content [49] makes a phage analysis more difficult. The first priority must be the contribution of new large amounts of data for phages infecting bacteria [12].

In addition, there is an urgent need for novel therapies to treat and prevent mastitis [50]. Bacteriophage therapy is an alternative to the antibiotic treatment of bovine mastitis [51], with a high specificity and a low probability for bacterial resistance development [52]. Many studies have demonstrated the effectiveness of bacteriophages in a variety of animal models to fight several mastitis-causing pathogenic bacteria. Some studies have shown how virulent phages such as SPW and SA phages are active against bovine mastitis-associated *S. aureus.* Moreover, SAJK-IND and MSP phages have specific lytic activity against several strains of *S. aureus* isolated from mastitis milk samples [53]. Indeed, mouse-induced mastitis models decreased their bacterial counts after treatment with a vBSM-A1 and vBSP-A2 phage cocktail [54]. Finally, several temperate phage mixtures have been shown to be more effective than using a single temperate phage for inhibiting *S. aureus.* According to the data obtained for the different models of mastitis, phage therapy using bacteriophages in this study can be considered an innovative alternative to antibiotics for the treatment of mastitis caused by *S. aureus*.

Finally, the proteomic analysis by LC-ESI-MS/MS performed in this study provides relevant insights into the search for potential phage origin diagnostic peptide biomarkers for mastitis-causing *S. aureus*. In addition, this method may be useful for searching peptide biomarkers for the identification and characterization of mastitis-causing species and for finding new *S. aureus* phages useful as possible therapies for mastitis.

## Figures and Tables

**Table 1:** Phage origin peptides identified in *Staphylococcus* *aureus* strains. NCBI (National Center for Biotechnology Information).

Strain	Protein	Peptide	Bacteria with 100% Homology Based on the NCBI Protein Database	Phages with 100% Homology Based on the NCBI Protein Database
S4	Uncharacterized phage protein	IRLPYYDVK	*Staphylococcus aureus*	*Staphylococcus* phage StauST398-2
S4	Uncharacterized phage protein	AVAELLKEINR	*Staphylococcus argenteus* *Staphylococcus simiae* *Staphylococcus aureus*	*Staphylococcus* virus 71 *Staphylococcus* virus 55 *Staphylococcus* virus 88
S4	Major capsid protein	LLHALPTGNDSGGDKLLPK	*Staphylococcus aureus* *Staphylococcus xylosus* *Staphylococcus muscae* *Staphylococcus haemolyticus* *Staphylococcus argenteus* *Streptococcus pneumoniae*	*Staphylococcus* phage phiSa2wa_st72 *Staphylococcus* phage phiSa2wa_st121mssa *Staphylococcus* phage vB_SauS_phi2 *Staphylococcus* phage StauST398-2 *Staphylococcus* phage LH1 *Staphylococcus* phage phiSa2wa_st30 *Staphylococcus* virus phi12 *Staphylococcus* virus 3a *Staphylococcus* virus phiSLT *Staphylococcus* phage tp310-2 *Staphylococcus* phage vB_SauS_JS02 *Staphylococcus* phage R4 *Staphylococcus* phage vB_SauS_fPfSau02 *Staphylococcus* phage SA137ruMSSAST121PVL
S4	Major capsid protein	RVSYTLDDDDFITDVETAKELKL	*Staphylococcus aureus* 12S01399 *Staphylococcus aureus* *Staphylococcus aureus* A9299 *Staphylococcus aureus* A9765 *Staphylococcus argenteus* *Staphylococcus aureus A6300* *Staphylococcus* sp. *Terrabacteria* group *Escherichia coli*	*Staphylococcus* phage LH1 *Staphylococcus* phage StauST398-2 *Staphylococcus* phage vB_SauS_phi2 *Staphylococcus* phage R4
S7	Major tail protein	LYVGVFNPEATK	*Staphylococcus aureus*	*Staphylococcus phage vB_SauS_ phi2* *Staphylococcus* virus phi12 *Staphylococcus* virus phiSLT *Staphylococcus* phage R4 *Staphylococcus* phage vB_SauS_JS02 *Staphylococcus* phage SH-St 15644 *Staphylococcus* virus 3a *Staphylococcus* phage P240
S8	Uncharacterized phage protein	M*NDSNQGLQANPQYTIHYLSQEITR	*Staphylococcus aureus*	*Staphylococcus* phage phiN315
S8	Major tail protein	AYINITGLGFAK	*Staphylococcus aureus* *Staphylococcus argenteus* *Pararheinheimera mesophila*	*Staphylococcus* phage phiNM3 *Staphylococcus* phage P282 *Staphylococcus* phage StauST398-4 *Staphylococcus* phage phiN315 *Staphylococcus* phage phi7247PVL *Staphylococcus* phage phiSa2wa_st22 *Staphylococcus* virus 77 *Staphylococcus* phage P954
S9	Major capsid protein	IYDRNSDTLDGLPVVNLK	*Staphylococcus aureus* *Staphylococcus argenteus*	*Staphylococcus* virus 85 *Staphylococcus* phage SP5 *Staphylococcus* virus phiETA2 *Staphylococcus* phage phiNM2 *Staphylococcus* virus SAP26 *Staphylococcus* phage SA12 *Staphylococcus* virus Baq Sau1
S11 and S20	Phage repressor, Cro/CI family	ELAEAIGVSQPTVSNWIQQTK	*Staphylococcus aureus* *Staphylococcus argenteus* *Staphylococcus sciuri*	*Staphylococcus* virus IPLA35 *Staphylococcus* phage SMSAP5 *Staphylococcus* phage vB_SauS_phi2
S11 and S20	Phage repressor, Cro/CI family	IQQLADYFNVPK	*Staphylococcus aureus* *Staphylococcus sciuri* *Staphylococcus pseudintermedius* *Staphylococcus devriesei* *Staphylococcus warneri* *Staphylococcus capitis* *Staphylococcus argenteus*	*Staphylococcus* phage SMSAP5 *Staphylococcus* phage vB_SauS_phi2 *Staphylococcus* virus IPLA35
S12 S10 and S14	Complement inhibitor	IYNEIDEALKSK	*Staphylococcus aureus, Enterobacter* sp. IF2SW-B1 *Klebsiella pneumoniae*	*Staphylococcus* phage 13 *Staphylococcus* phage phiNM3 *Staphylococcus* phage StauST398-1
S20	Major capsid protein	VSYTLDDDDFITDVETAK	*Staphylococcus aureus* *Staphylococcus haemolyticus* *Staphylococcus saprophyticus* *Staphylococcus warneri* *Staphylococcus argenteus* *Streptococcus pneumoniae* *Staphylococcus sciuri*	*Staphylococcus* phage phiSa2wa_st72 *Staphylococcus* phage tp310-2 *Staphylococcus* phage phiSa2wa_st121mssa *Staphylococcus* phage vB_SauS_phi2 *Staphylococcus* phage StauST398-2 *Staphylococcus* virus 3a *Staphylococcus* phage LH1 *Staphylococcus* phage phiSa2wa_st30 *Staphylococcus* virus phi12 *Staphylococcus* virus phiSLT *Staphylococcus* phage vB_SauS_JS02 *Staphylococcus* phage R4 *Staphylococcus* phage vB_SauS_fPfSau02 *Staphylococcus* phage SA137ruMSSAST121PVL
S20	Phage protein (DUF2479 domain)	SIINGKLDSQWTVPNEHK	*Staphylococcus aureus*	*Staphylococcus* phage DW2 *Staphylococcus* virus IPLA88
S18	N-acetylmuramoyl-L-alanine amidase	KEAGNYTVANVK	*Bacilli, Staphylococcus argenteus Staphylococcus aureus Staphylococcus* sp. HMSC34H10	*Staphylococcus* phage tp310-1 *Staphylococcus* phage tp310-2 *Staphylococcus* phage phi2958PVL *Staphylococcus* phage PVL *Staphylococcus* phage SA137ruMSSAST121PVL *Staphylococcus* virus IPLA35
S4	Phage protein NrdI	VETFLENETNQNNLIAVM*SSGNRNWGTNFAIAGDTISK	*Staphylococcus haemolyticus Staphylococcus hominis* *Staphyloccus aureus Staphylococcus aureus* subsp. *aureus* Z172	
S12	Complement inhibitor	IYNEIDEALK	*Staphylococcus. Aureus* *Klebsiella pneumoniae* *Enterobacter* sp. IF2SW-B1	*Staphylococcus* phage StauST398-1 *Staphylococcus* virus 13
S10	Complement inhibitor	IYNEIDEALKSKY	*Staphylococcus. aureus* *Klebsiella pneumoniae* *Enterobacter* sp. IF2SW-B2	*Staphylococcus* phage StauST398-1 *Staphylococcus* virus 13
S10	DDE-type integrase/transposase/recombinase	PC*PALM*NKRNSIATIHR	*Staphylococcus aureus*	
S9	DNA primase phage-associated	LLHHFYNPENTTALSFNDLNDKFKPANLQGKLVNIAD	*Staphylococcus aureus, Staphylococcus haemolyticus Staphylococcus capiti, Staphylococcus epidermidis Staphylococcus warneri Staphylococcus* sp. *HMSC077D08 Corynebacterium propinquum, Staphylococcus* sp. U *Staphylococcus lugdunensis Staphylococcus* sp. HMSC077B09	Uncultured Caudovirales Phage
S2	Phage repressor, Cro/CI family	AAHLEGELTDDEWQR	*Staphylococcus haemolyticus* *Staphylococcus warneri Staphylococcus agnetis, Staphylococcus chromogenes Staphylococcus haemolyticus Staphylococcus* sp. 58-22 *Staphylococcus capitis Staphylococcus pasteuri* *Bacillales Staphylococcus chromogenes Staphylococcus agnetis* *Escherichia coli, Staphylococcus aureus* 08-02906 *Staphylococcus aureus* VET0383R, *Staphylococcus aureus* VET0098R *Staphylococcus aureus M1487 Staphylococcus aureus, Staphylococcus aureus A6300 Staphylococcus aureus* subsp. *aureus* str. Newman *Staphylococcus aureus subsp. aureus* WBG10049, *Staphylococcus aureus* A9635, *Staphylococcus aureus* subsp. *aureus* MN8	*Staphylococcus* virus 71 *Staphylococcus* phage phiSa2wa_st1 *Staphylococcus* phage phiSa2wa_st5 *Staphylococcus* phage Henu2 *Staphylococcus* phage ROSA *Staphylococcus* phage phi7401PVL
S2	Phage repressor, Cro/CI family	VLDYADYIR	*Staphylococcus aureus Staphylococcus epidermidis* *Staphylococcus warneri* *Staphylococcus agnetis Staphylococcus warneri Staphylococcus chromogenes*, *staphylococcus* spp. *Staphylococcus schleiferi Staphylococcus simulans Staphylococcus haemolyticus, Staphylococcus pettenkoferi Staphylococcus lugdunensis Escherichia coli*	*Staphylococcus* virus 71 *Staphylococcus* phage phiSa2wa_st1 *Staphylococcus* phage phiSa2wa_st5 *Staphylococcus* phage Henu2 *Staphylococcus* phage ROSA *Staphylococcus* phage phi7401PVL
S9	DNA-binding protein	SLDNM*SLK	*Striga asiática* *Staphylococcus aureus* subsp. *aureus* 112808A *Staphylococcus aureus* A8819 *Staphylococcus argenteus* *Staphylococcus* spp. *Pseudomonas aeruginosa* *Flectobacillus* sp. BAB-3569 *Eoetvoesia caeni* *Arabidopsis thaliana*, *Coxiellaceae bacterium*, *Clostridia bacterium*	*Staphylococcus* phage vB_SauS_phi2
S19	DUF2479, Phage tail fiber, BppU family phage baseplate upper protein	HAGYVRC*KLF	*Staphylococcus aureus*, *Staphylococcus* sp. HMSC055H07 *Staphylococcus argenteus, Staphylococcus* sp. KY49P *Staphylococcus* sp. *HMSC035F11 Pseudomonas aeruginosa Escherichia coli*	*Staphylococcus* phage SA97 *Staphylococcus* virus 55 uncultured Caudovirales phage *Staphylococcus* virus 85 *Staphylococcus* virus 80 *Staphylococcus* virus phiETA3 *Staphylococcus* virus phiETA2 *Staphylococcus* phage 55-2 *Staphylococcus* phage B166 *Staphylococcus* phage B236 *Staphylococcus* virus SAP26 *Staphylococcus* virus 88 *Staphylococcus* virus phiETA *Staphylococcus* virus 11 *Staphylococcus* phage SP5 *Staphylococcus* virus 69 *Staphylococcus* phage ROSA *Staphylococcus* phage TEM123 *Staphylococcus* virus 92 *Staphylococcus* phage StauST398-1 *Staphylococcus* virus phiNM2 *Staphylococcus* virus phiNM1 *Staphylococcus* virus 29 *Staphylococcus* phage vB_SauS-SAP27 *Staphylococcus* virus 80alpha *Staphylococcus* phage HSA84 *Staphylococcus* virus phiMR11 *Staphylococcus* phage SAP33 *Staphylococcus* phage 3MRA
S12	Phage protein (DUF4393 domain)	NSPIDLNSTEISLNNLER	*Staphylococcus aureus* *Staphylococcus* spp. *Staphylococcus argenteus*	*Staphylococcus* phage StauST398-1
S12	Phage protein (DUF669 domain)	MNFNLNLQGAQELGN	*Staphylococcus capitis* *Staphylococcus epidermidis Staphylococcus caprae Staphylococcus devriesei Staphylococcus warneri*	*Staphylococcus* virus phiMR11
S10	GNAT family N-acetyltransferase	IINYARQNNYESLLTSIVSNNIGAK	*Staphylococcus aureus Staphylococcus aureus* subsp. *anaerobius* *Staphylococcus aureus* subsp. *aureus* Mu50 *Staphylococcus hominis* *Escherichia coli*	
S5	Holin, phage phi LC3 family	SQDSNLTPELSTKAPK	*Staphylococcus aureus*	*Staphylococcus* phage HSA84 *Staphylococcus* phage SP5
S6	ImmA/IrrE family metallo-endopeptidase	EKAKIFGDFDMNDSGVYDEENSTIIYNPLDSITR	*Staphylococcus aureus* subsp. *aureus* H19 *Staphylococcus aureus* *Staphylococcus aureus* subsp. *aureus* *Staphylococcus aureus subsp. aureus 21204*	
S16	Involved in the expression of fibrinogen-binding protein phage-associated	ESINANTYINQNLEK	*Staphylococcus aureus*	
S16	Involved in the expression of fibrinogen-binding protein phage-associated	VAVLSTPLVTSFESK	*Staphylococcus aureus*	
S17	N-6 DNA methylase; N6_Mtase domain-containing protein	KDGEILFDAIDIYLRNK	*Staphylococcus aureus*	*Staphylococcus* phage phi-42
S4	Phage DNA-binding protein	GDM*FVVITIM*MQQIK	*Staphylococcus aureus* *Staphylococcus warneri*	
S9	Phage terminase	KLYIIEEYVKQGM	*Staphylococcus aureus Staphylococcus argenteus Staphylococcus* sp. HMSC58E11 *Allobacillus* sp. SKP4-8	*Staphylococcus* virus Baq_Sau1 *Staphylococcus* virus phiETA2 *Staphylococcus* virus 69 *Staphylococcus* virus 11 *Staphylococcus* virus 80alpha
S14	Integrase	M*PVYKDGNTGKWYFSI	*Staphylococcus aureus*	*Staphylococcus* phage B166 *Staphylococcus* virus phiMR25 *Staphylococcus* virus 88
S4	Phage repressor	ISKVQQLADYFNVPK	*Staphylococcus aureus, Staphylococcus chromogenes* *Staphylococcus hyicus*	*Staphylococcus* virus 80
S13	Toxin Phage protein; Pathogenicity island protein	NLDGVWLGDLILIKRGLSDR	*Staphylococcus aureus, Staphylococcus* sp. HMSC58E11, *Staphylococcus argenteus, Escherichia coli*	*Staphylococcus* phage phiSa2wa_st80 *Staphylococcus* phage 3MRA *Staphylococcus* phage phiSa2wa_st5
S16	Toxin Phage protein; Pathogenicity island protein	SDREKAGILFEELAHNK	*Staphylococcus aureus* *Escherichia coli* *Staphylococcus argenteus Staphylococcus* sp. HMSC58E11	*Staphylococcus* phage 3MRA *Staphylococcus* phage phiSa2wa_st5 *Staphylococcus* phage phiSa2wa_st80 *Staphylococcus* phage phiJB *Staphylococcus* phage phi7401PVL
S6	PBSX family phage terminase	QADNTYVHHSTYLNNPFISKQFIQEAESAKQR	*Staphylococccus* spp.	
S11	PBSX family phage terminase	QGVSHLFKVTKSPM*R	*Staphylococcus aureus Staphylococcus lentus Staphylococcus sciuri*	
S20	Phage-related cell wall hydrolase; Peptidase C51; CHAP domain-	EVPNEPDYIVIDVC*EDYSASK	*Staphylococcus argenteus* *Staphylococcus* sp. HMSC36F05	*Staphylococcus* virus IPLA88 *Staphylococcus* virus phiNM2 *Staphylococcus* phage SAP40 *Staphylococcus* phage phi 53 *Staphylococcus* virus phiNM4 *Staphylococcus* phage SA12 *Staphylococcus* virus 69 *Staphylococcus* phage SA97 *Staphylococcus* phage TEM123 *Staphylococcus* virus 11 *Staphylococcus* virus phiMR25 *Staphylococcus* virus 53 *Staphylococcus* phage SAP33
S5	Phage antirepressor Ant	QDWLAM*EVLPAIR	*Staphylococcus aureus, Staphylococcus simulans Staphylococcus argenteus Staphylococcus pseudintermedius*	*Staphylococcus* phage SA75 *Staphylococcus* phage SA13
S11	Phage capsid protein	M*AEETNSNVTEETEVNE	*Staphylococcus, aureus Staphylococcus* spp.	
S4	Phage encoded lipoprotein	IHDKELDDPSEEESKLTQEEENSI	*Staphylococcus aureus, Staphylococcus capitis, Staphylococcus epidermidis, Staphylococcus cohnii, Staphylococcus haemolyticus*	*Staphylococcus* phage SPbeta-like
S2	Phage head morphogenesis protein	KDVQRIVSHVT	*Staphylococcus aureus* *Staphylococcus argenteus*	
S9	YhgE/Pip, Phage infection protein	LNEYM*PNIEKLLNVASNDIPAQFPK	*Staphylococcusaureus, Staphylococcus haemolyticus Staphylococcus* sp. HMSC34C02	
S14	Minor structural protein	KTTSEALKEVLSDT	*Staphylococcus aureus*	
S4	Phage portal protein	EPKPVDATGADDPLKPDDRM*ITNFHANLVDQKVSY	*Staphylococcus aureus*	
S5	Phage protein	VHISEFKYPLYM*DFLGTKGELE	*Staphylococcusaureus* *Staphylococcus haemolyticus*	
S15	Phage protein	MSHNALTTGIGIGAGAG	*Staphylococcus aureus*	
S2	Phage protein	EITDGEISSVLTM*M*	*Staphylococcus aureus, Staphylococcus hominis Staphylococcus epidermidis*	
S20	Phage recombination protein Bet	KSSTTYEVNGETVK	*Staphylococcus aureus, Staphylococcus sciuri*	
S2	Phage resistance protein	ESVDTGEITANTTRTVK	*Staphylococcus aureus Staphylococcus fleurettii* *Staphylococcus pasteuri* *Staphylococcus epidermidis Staphylococcus warneri Staphylococcus schleiferi Escherichia coli*	
S13	Tail tape measure protein	GM*PTGTNVYAVKGGIADK	*Staphylococcus aureus, Staphylococcus saprophyticus, Staphylococcus pseudoxylosus*	*Staphylococcus phage phiSa2wa_st5 Staphylococcus phage phi3A Staphylococcus phage SH-St 15,644 Staphylococcus virus 3a*
S3	Tail tape measure protein	VQHPGKLVNKVM*SGLNINFGGGANATAK	*Staphylococcus aureus*	
S4	Tail tape measure protein	QM*MEGLSGVMDLAAVSGEDLGAVSDIVTDGLTAFGLKAKDSG	*Staphylococcus aureus*	
S2	Tail tape measure protein	AEEAGVTVKQL	*Staphylococcus aureus* *Staphylococcus cohnii* *Staphylococcus sp. HMSC061H04* *Staphylococcus hominis* *Staphylococcus capitis* *Staphylococcus cohnii* *Staphylococcus* sp. *HMSC061H04* *Staphylococcus* sp. *HMSC067G10* *Staphylococcus* *Staphylococcus haemolyticus* *Enterococcus faecium* *Staphylococcus epidermidis* *Staphylococcus* sp. *HMSC067G10* *Staphylococcus haemolyticus* *Enterococcus faecium* *Staphylococcus epidermidis*	*Staphylococcus* phage SPbeta-like
S10	Phage repressor, Cro/CI family	QKNVLNYANEQLDEQNKV	*Staphylococcus aureus, Bacilli, Staphylococcus hyicus Staphylococcus epidermidis*	*Staphylococcus virus phiNM2 Staphylococcus virus 53 Staphylococcus virus 80alpha*
S13	Phage protein	KSNVEAFSNAVK	*Staphylococcus aureus*	*Staphylococcus* virus 80alpha *Staphylococcus* virus phiNM1 *Staphylococcus* virus phiNM2
S11	Phage protein	PYHDLSDERIM*EELKK	*Staphylococcus aureus Staphylococcus argenteus taphylococcus schweitzeri*	*Staphylococcus* virus phiETA2 *Staphylococcus* phage P630 *Staphylococcus* virus SAP26 *Staphylococcus* phage B236 *Staphylococcus* virus 88 *Staphylococcus* prophage phiPV83
S4	Minor structural protein	LNDNISNINTIV	*Pseudomonas aeruginosa* *E. coli* *Pararheinheimera mesophila* *Staphylococcus pseudintermedius Staphylococcus epidermidis, Staphylococcus* sp. KY49P *Staphylococcus argenteus Staphylococcus schleiferi Staphylococcus hyicus Staphylococcus* sp. *HMSC063H12 Staphylococcus aureus*	*Staphylococcus* virus 77 *Staphylococcus* phage P630 *Staphylococcus* phage SA780ruMSSAST101 *Staphylococcus* phage phiSa119 *Staphylococcus* phage phiN315 *Staphylococcus* phage SA7 *Staphylococcus* phage JS01 *Staphylococcus* phage StauST398-4 *Staphylococcus* virus 13 *Staphylococcus* phage 23MRA *Staphylococcus* virus 108PVL *Staphylococcus* phage phiBU01 *Staphylococcus* phage PVL *Staphylococcus* phage tp310-1 *Staphylococcus* phage P954 *Staphylococcus* phage SA345ruMSSAST8 *Staphylococcus* phage phiNM3 *Staphylococcus* virus 77 *Staphylococcus* phage phiSa2wa_st22 *Staphylococcus* phage SA1014ruMSSAST7 *Staphylococcus* phage P282 *Staphylococcus* prophage phiPV83 *Staphylococcus* phage 3 AJ-2017 *Staphylococcus* phage SAP090B *Staphylococcus* phage IME1346_01 *Staphylococcus* phage phi5967PVL *Staphylococcus* phage P1105 *Staphylococcus* phage IME1361_01
S9	PhiETA ORF58-like protein	GMVASMQMQVVQVNVLTM*ELAQQNAMLTQQLTELK	*Staphylococcus aureus*	
S4	Phage portal protein	TEQLPRLEML	*Staphylococcus* aureus, *Staphylococcus* sp. HMSC063A07, *Staphylococcus* lugdunensis, *Staphylococcus* sp. HMSC068D08, *Staphylococcus* sp. HMSC069E09	
S4	Prophage, terminase	KDRYSSVSY	*Staphylococcus aureus, Staphylococcus delphini, Staphylococcus pseudintermedius, Staphylococcus agnetis, Staphylococcus epidermidis, Staphylococcus hominis, Staphylococcus haemolyticus, Paenibacillus sophorae*	*Staphylococcus* phage SPbeta-like
S4	Prophage tail domain; Peptidase	VLEM*IFLGEDPK	*Staphylococcus aureus* *E. coli* *Bacilli*	*Staphylococcus* phage phi7401PVL *Staphylococcus* phage phiSa2wa_st121mssa *Staphylococcus* virus 3a *Staphylococcus* virus phiSLT *Staphylococcus* phage tp310-2 *Staphylococcus* phage SA137ruMSSAST121PVL *Staphylococcus* phage phiSa2wa_st5 *Staphylococcus* phage phiSa2wa_st1 *Staphylococcus* phage SH-St 15644 *Staphylococcus* phage phi2958PVL *Staphylococcus* virus IPLA35 *Staphylococcus* phage P240 *Staphylococcus* phage vB_SauS_JS02 *Staphylococcus* virus 42e *Staphylococcus* virus phi12 *Staphylococcus* phage phiSa2wa_st72 *Staphylococcus* phage vB_SauS_fPfSau02 *Staphylococcus* phage phiSa2wa_st30 *Staphylococcus* phage vB_SauS_phi2 *Staphylococcus* phage StauST398-2
S15	Site-specific integrase	VEELEDSEIHKK	*Staphylococcus aureus, Staphylococcus epidermidis Staphylococcus haemolyticus Staphylococcus condimenti Staphylococcus* sp. HMSC035D11 *Staphylococcus warneri*	uncultured *Caudovirales* phage Sequence ID: ASN72447.1
S13	Site-specific integrase	KEAGSIINLHTINNALKSAC*R	*Staphylococcus* aureus *Staphylococcus* sp.	
S6	Site-specific integrase	YLNRNFVFTNHK	*Staphylococcus aureus, Staphylococcus argenteus Staphylococcus cohini Staphylococcus lugdunensis* *Staphylococcus caeli Staphylococcus* sp. 47.1	
S9	Terminase large subunit	KAMIKASPK	*Staphylococcusaureus* *Escherichia coli* *Staphylococcus* sp. HMSC74F04 *Staphylococcus* sp. HMSC055H07 *Cutibacterium acnes Staphylococcus warneri Brevibacillus laterosporus* *Bacillus cihuensis* *Paenibacillus larvae*	*Staphylococcus* phage vB_SauS_JS02 *Staphylococcus* phage *Staphylococcus* phage phiSa2wa_st5 *Staphylococcus* phage LH1 *Staphylococcus* phage phiSa2wa_st1 *Staphylococcus* phage phiSa2wa_st121mssa *Staphylococcus* virus IPLA35 *Staphylococcus* phage tp310-2 *Staphylococcus* virus phiSLT *Staphylococcus* phage StauST398-2 *Staphylococcus* phage vB_SauS_phi2 *Staphylococcus* virus phi12 *Staphylococcus* phage SMSAP5 *Staphylococcus* phage phi2958PVL *Staphylococcus* virus 3a *Staphylococcus* phage YMC/09/04/R1988
S20	Phage repressor, Cro/CI family	RIQQLADYFNVPK	*Staphylococcus aureus Staphylococcus pettenkoferi Staphylococcus pettenkoferi Staphylococcus capitis Staphylococcus devriesei*	*Staphylococcus* phage vB_SauS_phi2 *Staphylococcus* virus IPLA35
S4	Transposase B from transposon Tn554 O	WDRRNLPLPDDK	*Staphylococcus aureus*, *Staphylococcuspettenkoferi* *Staphylococcushominis*, *Quasibacillus thermotolerans* *Staphylococcaceae* *Staphylococcusvitulinus* *Streptococcus suis* *Staphylococcusfelis* *Salinicoccus roseus* *Staphylococcus epidermidis* *Staphylococcuslentus* *Staphylococcuswarneri* *Staphylococcusepidermidis* *Staphylococcuschromogenes* *Staphylococcus* sp. HMSC058E01 *Enterococcus faecium* *Staphylococcus epidermidis* VCU065 *Staphylococcus* *cohnii* *Negativicoccus succinicivorans* *Eubacteriaceae bacterium* *Staphylococcus* *Enterococcus faecium* *Enterococcus* *Staphylococcus fleurettii* *Staphylococcus* sp. 47.1 Bacilli *Staphylococcus* sp. SKL71207 Lactobacillales	
S13	Uncharacterized phage protein	C*VSGIAGGAVTGGTTLGLAGAG	*Staphylococcus aureus Staphylococcus argenteus* *Staphylococcus schweitzeri* *Staphylococcus schweitzeri* *Staphylococcus hyicus* *Staphylococcus agnetis*	
S13	Uncharacterized phage protein	DIITVYC*PENGTATDEY	*Staphylococcus aureus*	
S20	Uncharacterized phage protein	QTDVPSWVPM*VLR	*Staphylococcusaureus Staphylococcus* sp. HMSC74F04 Bacilli *Staphylococcus* *Staphylococcus* *argenteus* *Staphylococcus* sp. HMSC063H12	
S12	Uncharacterized phage protein	IIINHDEIDLL	*Staphylococcus aureus Staphylococcus epidermidis* *Staphylococcus hominis* *Staphylococcus haemolyticus* *Staphylococcus* sp. HMSC067G10 *Staphylococcus haemolyticus* *Staphylococcus epidermidis* *Staphylococcus petrasii* *Staphylococcus capitis*	*Staphylococcus* phage SPbeta-like
S14	Uncharacterized phage protein	TSIELITGFTK	*Staphylococcus aureus, Staphylococcus sciuri, Staphylococcus schweitzeri*, *Staphylococcus* spp.	*Staphylococcus* phage phi879, *Staphylococcus* phage phi575, *Staphylococcus* phage PVL, *Staphylococcus* prophage phiPV83, *Staphylococcus* phage SA45ruMSSAST97
S3	Uncharacterized phage protein	EFRNKLNELGADK	*Staphylococcusaureus, Streptococcus pneumoniae,* Terrabacteria group	*Staphylococcus* phage phi7401PVL, *Staphylococcus* phage tp310-2, *Staphylococcus* phage vB_SauS_phi2, *Staphylococcus* virus IPLA35, *Staphylococcus* phage phiSa2wa_st30, *Staphylococcus* virus 47, *Staphylococcus virus 3a*
S3	Phage repressor, Cro/CI family	HLEEVDIR	*Staphylococcusaureus, Paxillus involutus* ATCC 200175, *Brassica cretica*, *Staphylococcus* epidermidis, *Staphylococcus* spp., *Enterobacter hormaechei*	
S4	YhgE/Pip; Phage infection protein	APQSTSVKK	*Staphylococcusaureus*, *Staphylococcusschweitzeri, Staphylococcus* sp.	
S4	YhgE/Pip Phage infection protein	ALNFAADDVPAQFPK	*S. aureus*, *Staphylococcus* sp. HMSC36A10, *Staphylococcus* sp. HMSC34H10, *Pseudomonas aeruginosa*, *E. coli*	

**Table 2:** Phage biomarker peptides that belong to bacteriophages and phylogenomic tree clusters. Relationships between specific phage biomarker peptides and phylogenomic tree clusters.

Protein	Peptide	Phages	Cluster Located
Major capsid protein	VSYTLDDDDFITDVETAK	*Staphylococcus* phage phiSa2wa_st72 *Staphylococcus* phage tp310-2 *Staphylococcus* phage phiSa2wa_st121mssa *Staphylococcus* phage vB_SauS_phi2 *Staphylococcus* phage StauST398-2 *Staphylococcus* virus 3a *Staphylococcus* phage LH1 *Staphylococcus* phage phiSa2wa_st30 *Staphylococcus* virus phi12 *Staphylococcus* virus phiSLT *Staphylococcus* phage vB_SauS_JS02 *Staphylococcus* phage R4 *Staphylococcus* phage vB_SauS_fPfSau02 *Staphylococcus* phage SA137ruMSSAST121PVL	Cluster D
Major capsid protein	LLHALPTGNDSGGDKLLPK	*Staphylococcus* phage phiSa2wa_st72 *Staphylococcus* phage phiSa2wa_st121mssa *Staphylococcus* phage vB_SauS_phi2 *Staphylococcus* phage StauST398-2 *Staphylococcus* phage LH1 *Staphylococcus* phage phiSa2wa_st30 *Staphylococcus* virus phi12 *Staphylococcus* virus 3ª *Staphylococcus* virus phiSLT *Staphylococcus* phage tp310-2 *Staphylococcus* phage vB_SauS_JS02 *Staphylococcus* phage R4 *Staphylococcus* phage vB_SauS_fPfSau02 *Staphylococcus* phage SA137ruMSSAST121PVL	Cluster D
Major capsid protein	RVSYTLDDDDFITDVETAKELKL	*Staphylococcus* phage LH1 *Staphylococcus* phage StauST398-2 *Staphylococcus* phage vB_SauS_phi2 *Staphylococcus* phage R4	Cluster D
Major tail protein	LYVGVFNPEATK	*Staphylococcus* phage vB_SauS_ phi2 *Staphylococcus* virus phi12 *Staphylococcus* virus phiSLT *Staphylococcus* phage R4 *Staphylococcus* phage vB_SauS_JS02 *Staphylococcus* phage SH-St 15644 *Staphylococcus* virus 3a *Staphylococcus* phage P240	Cluster D
Phage repressor, Cro/CI family	ELAEAIGVSQPTVSNWIQQTK	*Staphylococcus* virus IPLA35 *Staphylococcus* phage SMSAP5 *Staphylococcus* phage vB_SauS_phi2	Cluster D
Phage repressor, Cro/CI family	IQQLADYFNVPK	*Staphylococcus* virus IPLA35 *Staphylococcus* phage SMSAP5 *Staphylococcus* phage vB_SauS_phi2	Cluster D
Major tail protein	AYINITGLGFAK	*Staphylococcus* phage phiNM3 *Staphylococcus* phage StauST398-4 *Staphylococcus* phage P282 *Staphylococcus* phage phiN315 *Staphylococcus* phage phi7247PVL *Staphylococcus* phage phiSa2wa_st22 *Staphylococcus* virus 77 *Staphylococcus* phage P954	Cluster B.1
Major capsid protein	IYDRNSDTLDGLPVVNLK	*Staphylococcus* virus 85 *Staphylococcus* phage SP5 *Staphylococcus* virus phiETA2 *Staphylococcus* phage phiNM *Staphylococcus* virus SAP26 *Staphylococcus* phage SA12 *Staphylococcus* virus Baq Sau1	Cluster A.1
Uncharacterized phage protein	AVAELLKEINR	*Staphylococcus* virus 71 *Staphylococcus* virus 55 *Staphylococcus* virus 88	Cluster A.2
DUF2479, Phage tail fiber, BppU family phage baseplate upper protein	HAGYVRCKLF	*Staphylococcus* phage SA97 *Staphylococcus* virus 55 uncultured Caudovirales phage *Staphylococcus* virus 85 *Staphylococcus* virus 80 *Staphylococcus* virus phiETA3 *Staphylococcus* virus phiETA2 *Staphylococcus* phage 55-2 *Staphylococcus* phage B166 *Staphylococcus* phage B236 *Staphylococcus* virus SAP26 *Staphylococcus* virus 88 *Staphylococcus* virus phiETA *Staphylococcus* virus 11 *Staphylococcus* phage SP5 *Staphylococcus* virus 69 *Staphylococcus* phage ROSA *Staphylococcus* phage TEM123 *Staphylococcus* virus 92 *Staphylococcus* phage StauST398-1 *Staphylococcus* virus phiNM2 *Staphylococcus* virus phiNM1 *Staphylococcus* virus 29 *Staphylococcus* phage vB_SauS-SAP27 *Staphylococcus* virus 80alpha *Staphylococcus* phage HSA84 *Staphylococcus* virus phiMR11 *Staphylococcus* phage SAP33 *Staphylococcus* phage 3MRA	Cluster A
Phage terminase	KLYIIEEYVKQGM	*Staphylococcus* virus Baq_Sau1 *Staphylococcus* virus phiETA2 *Staphylococcus* virus 69 *Staphylococcus* virus 11 *Staphylococcus* virus 80alpha	Cluster A.1
Phage-related cell wall hydrolase; Peptidase C51; CHAP domain-	EVPNEPDYIVIDVC*EDYSASK	*Staphylococcus* virus IPLA88 *Staphylococcus* virus phiNM2 *Staphylococcus* phage SAP40 *Staphylococcus* phage phi 53 *Staphylococcus* virus phiNM4 *Staphylococcus* phage SA12 *Staphylococcus* virus 69 *Staphylococcus* phage SA97 *Staphylococcus* phage TEM123 *Staphylococcus* virus 11 *Staphylococcus* virus phiMR25 *Staphylococcus* virus 53 *Staphylococcus* phage SAP33	Cluster A.1
Prophage_tail domain-; Peptidase	VLEM*IFLGEDPK	*Staphylococcus* phage phi7401PVL *Staphylococcus* phage phiSa2wa_st121mssa *Staphylococcus* virus 3a *Staphylococcus* virus phiSLT *Staphylococcus* phage tp310-2 *Staphylococcus* phage SA137ruMSSAST121PVL *Staphylococcus* phage phiSa2wa_st5 *Staphylococcus* phage phiSa2wa_st1 *Staphylococcus* phage SH-St 15644 *Staphylococcus* phage phi2958PVL *Staphylococcus* virus IPLA35 *Staphylococcus* phage P240 *Staphylococcus* phage vB_SauS_JS02 *Staphylococcus* virus 42e *Staphylococcus* virus phi12 *Staphylococcus* phage phiSa2wa_st72 *Staphylococcus* phage vB_SauS_fPfSau02 *Staphylococcus* phage phiSa2wa_st30 *Staphylococcus* phage vB_SauS_phi2 *Staphylococcus* phage StauST398-2	Cluster D
Terminase large subunit	KAM*IKASPK	*Staphylococcus* phage vB_SauS_JS02 *Staphylococcus* phage *Staphylococcus* phage phiSa2wa_st5 *Staphylococcus* phage LH1 *Staphylococcus* phage phiSa2wa_st1 *Staphylococcus* phage phiSa2wa_st121mssa *Staphylococcus* virus IPLA35 *Staphylococcus* phage tp310-2 *Staphylococcus* virus phiSLT *Staphylococcus* phage StauST398-2 *Staphylococcus* phage vB_SauS_phi2 *Staphylococcus* virus phi12 *Staphylococcus* phage SMSAP5 *Staphylococcus* phage phi2958PVL *Staphylococcus* virus 3a *Staphylococcus* phage YMC/09/04/R1988	Cluster D
Uncharacterized phage protein	TSIELITGFTK	*Staphylococcus* phage phi879, *Staphylococcus* phage phi575, *Staphylococcus* phage PVL, *Staphylococcus* prophage phiPV83, *Staphylococcus* phage SA45ruMSSAST97	Cluster B2
Uncharacterized phage protein	EFRNKLNELGADK	*Staphylococcus* phage phi7401PVL, *Staphylococcus* phage tp310-2, *Staphylococcus* phage vB_SauS_phi2, *Staphylococcus* virus IPLA35, *Staphylococcus* phage phiSa2wa_st30, *Staphylococcus* virus 47, *Staphylococcus* virus 3a	Cluster D
Phage protein	KSNVEAFSNAVK	*Staphylococcus* virus 80alpha *Staphylococcus* virus phiNM1 *Staphylococcus* virus phiNM2	Cluster A.1
Phage repressor, Cro/CI family	QKNVLNYANEQLDEQNKV	*Staphylococcus* virus phiNM2 *Staphylococcus* virus 53 *Staphylococcus* virus 80alpha	Cluster A.1
Tail tape measure protein	GM*PTGTNVYAVKGGIADK	*Staphylococcus* phage phiSa2wa_st5 *Staphylococcus* phage phi3A *Staphylococcus* phage SH-St 15,644 *Staphylococcus* virus 3a	Cluster D
integrase	M*PVYKDGNTGKWYFSI	*Staphylococcus* phage B166 *Staphylococcus* virus phiMR25 *Staphylococcus* virus 88	Cluster A

## Data Availability

All relevant data are included in the article. The mass spectrometric data were deposited into the public database PRIDE (Proteomics Identification Database), with the dataset identifier PXD023530.

## Supplementary Materials

The following are available online at https://www.mdpi.com/article/10.3390/foods10040799/s1: Figure S1: MS/MS spectrums for *S. aureus*-specific peptide biomarkers. The corresponding peptides were tested for specificity using the BLASTp algorithm. Excel Dataset Supplemental Data 1: Complete nonredundant peptide dataset. Supplemental Data 2: Table S1: *Staphylococcus aureus* (SA) strains used in this study. Table S2: Linage, authors and accession number of studied bacteriophages [55,56,57,58,59,60,61,62,63,64,65,66,67,68,69,70,71,72,73,74,75,76,77,78,79,80,81,82,83,84,85,86,87,88].
