# Supplementary material for: Proteomic Characterization of Bacteriophage Peptides from the Mastitis Producer Staphylococcus aureus by LC-ESI-MS/MS and the Bacteriophage Phylogenomic Analysis
Source: Foods. 2021 Apr 8;10(4):799. doi: 10.3390/foods10040799 (PMC8068337; doi:10.3390/foods10040799)

IRLPYYDVK 389.5571 $[M+2H]^{2+}$

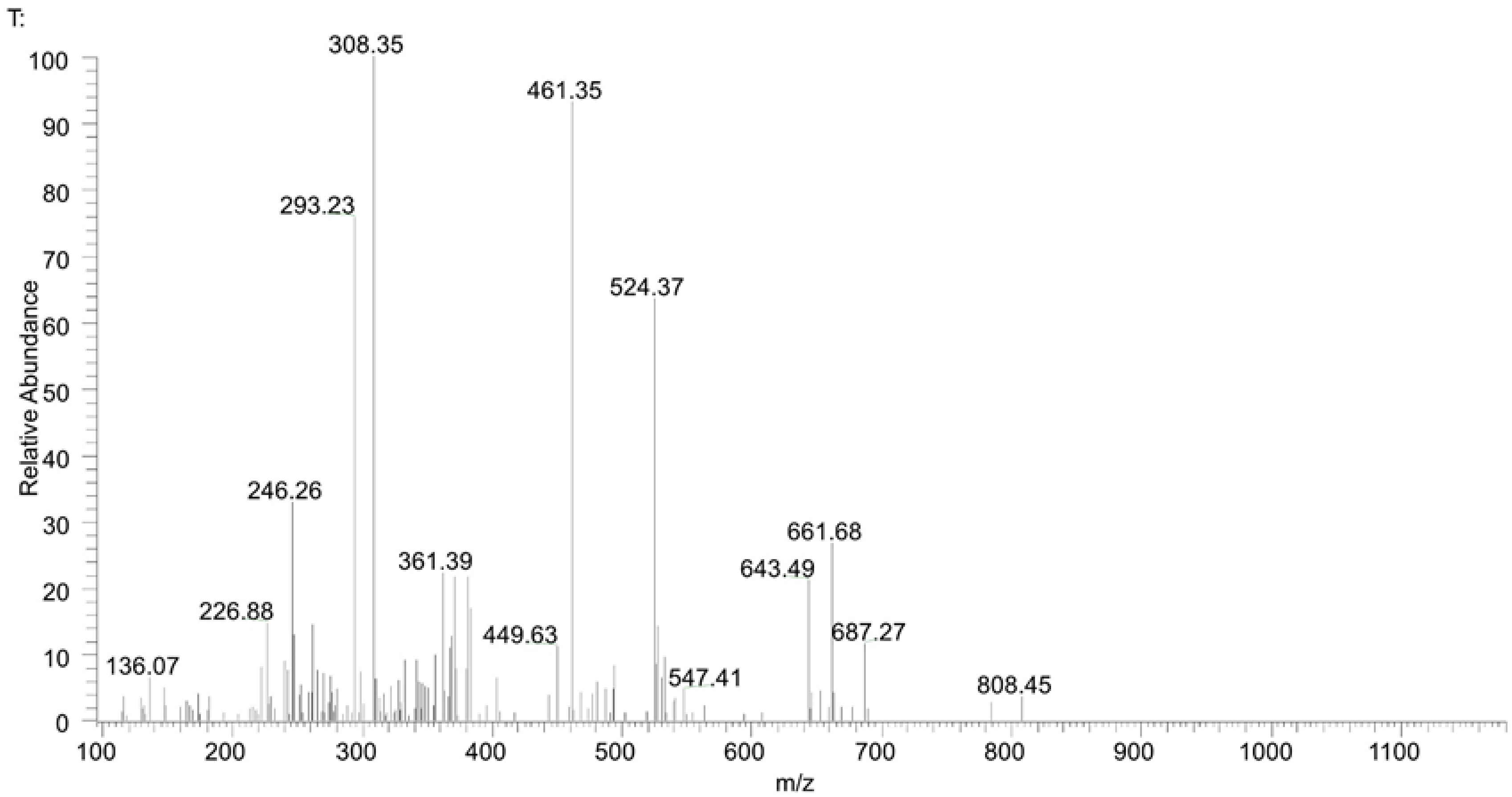

LYVGVFNPEATK 669.3586 [M+2H]$^{2+}$

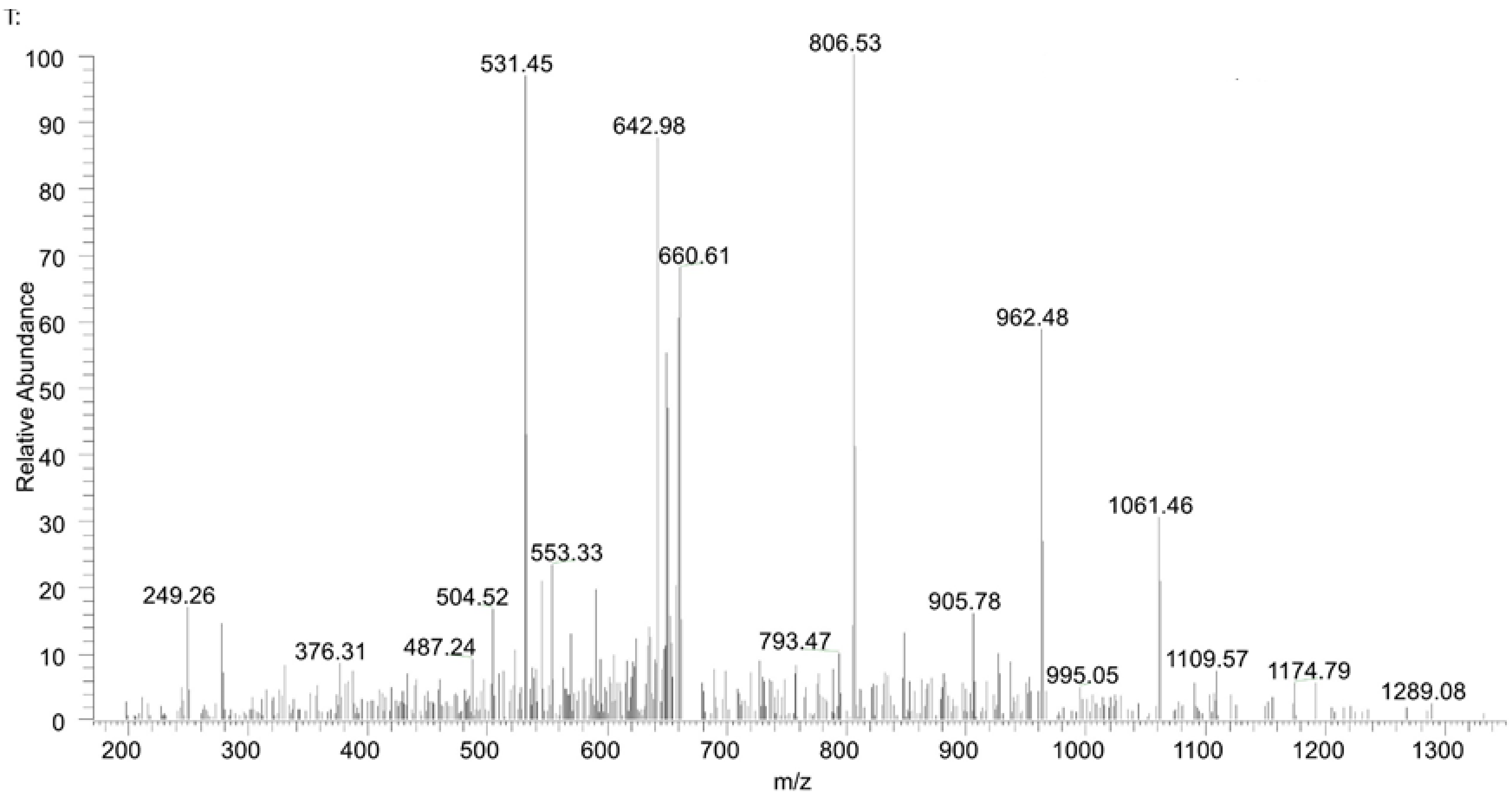

SIINGKLDSQWTVPNEHK 1033.5369 $[M+2H]^{2+}$

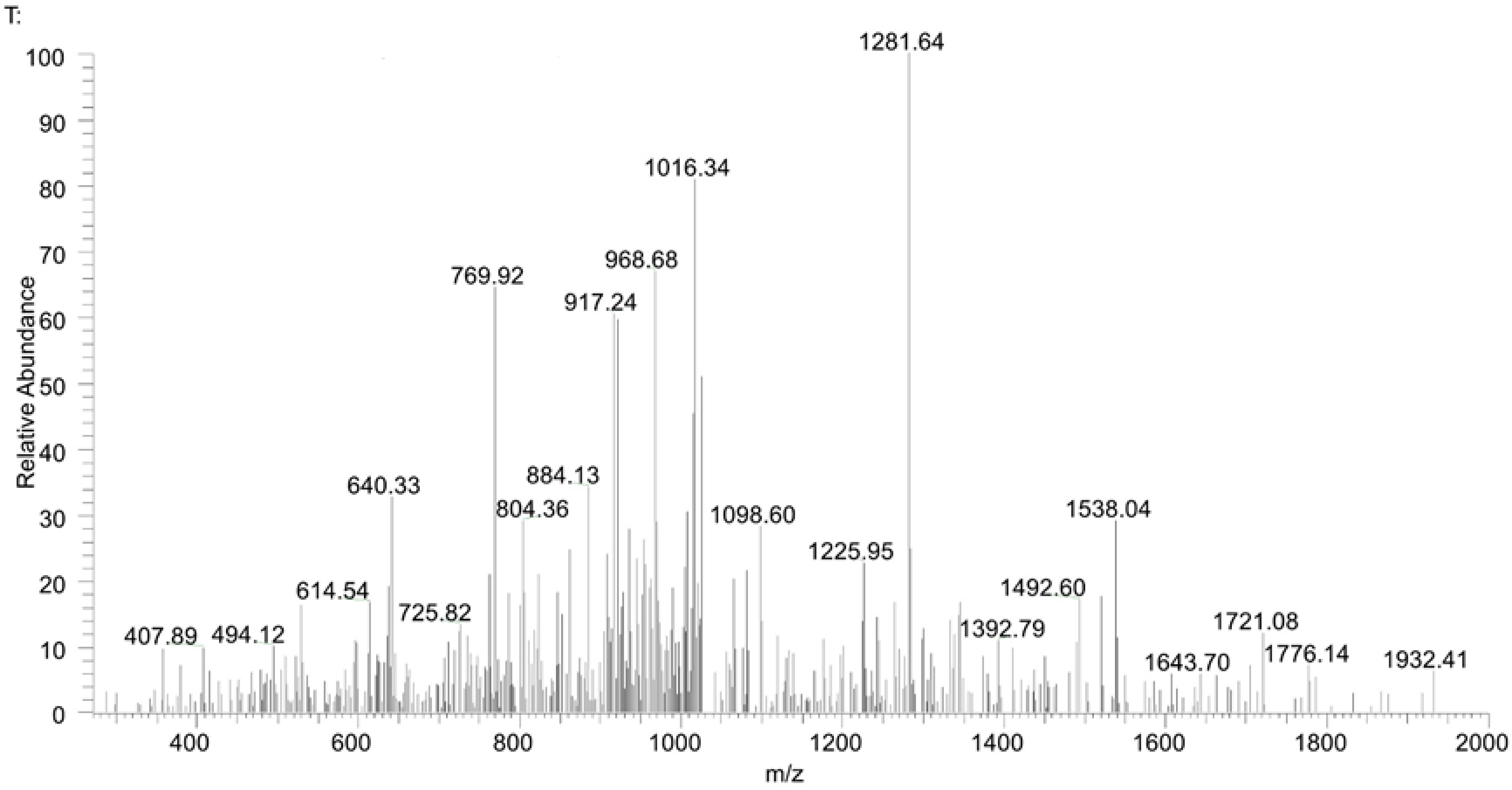

M[147.04]NDSNQGLQANPQYTIHYLSQEITR 979.7980 $[M+2H]^{2+}$

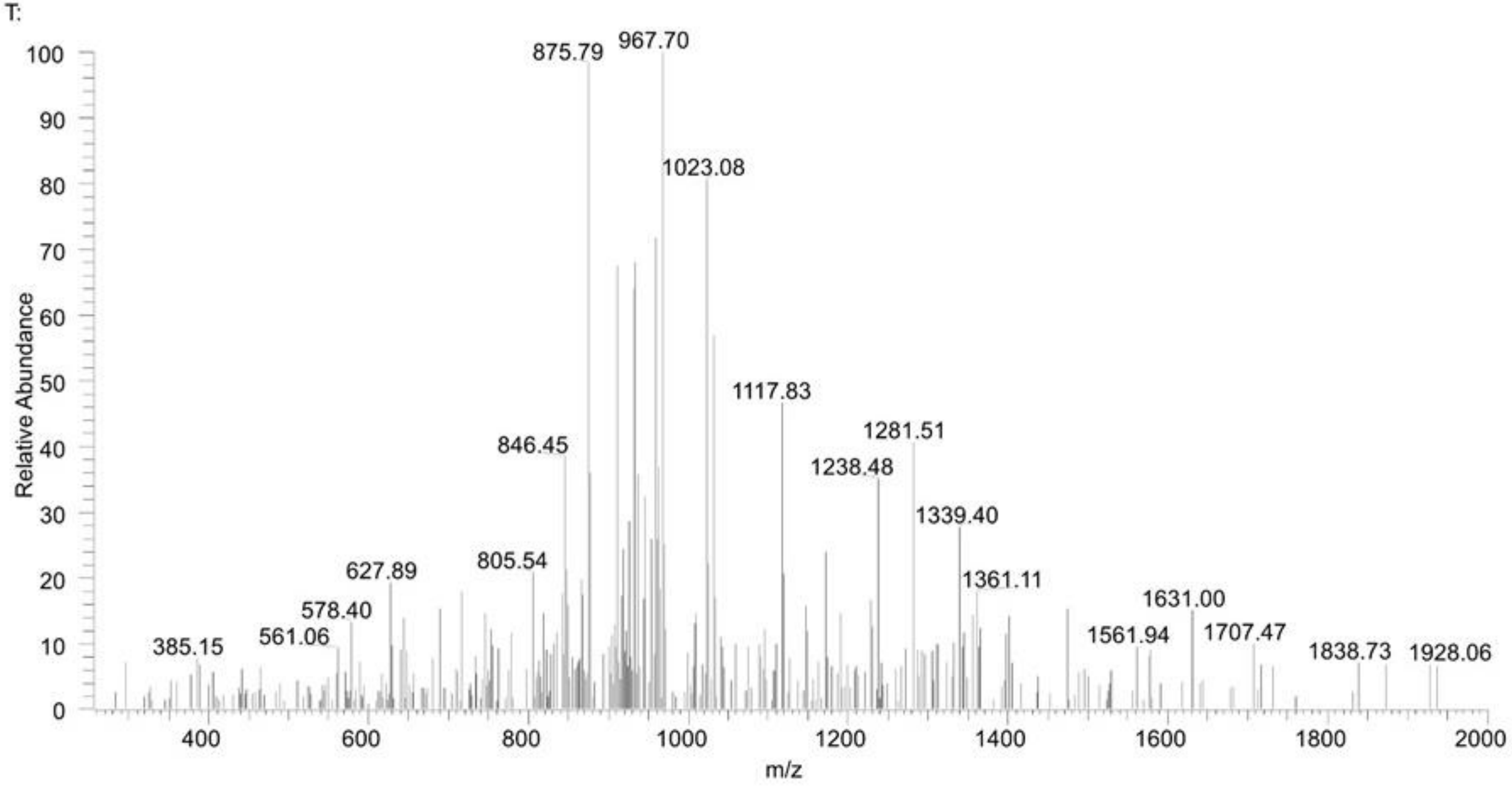

[42.01]PC[160.03]PALMNKRNSIATIHR 674.3538 $[M+2H]^{2+}$

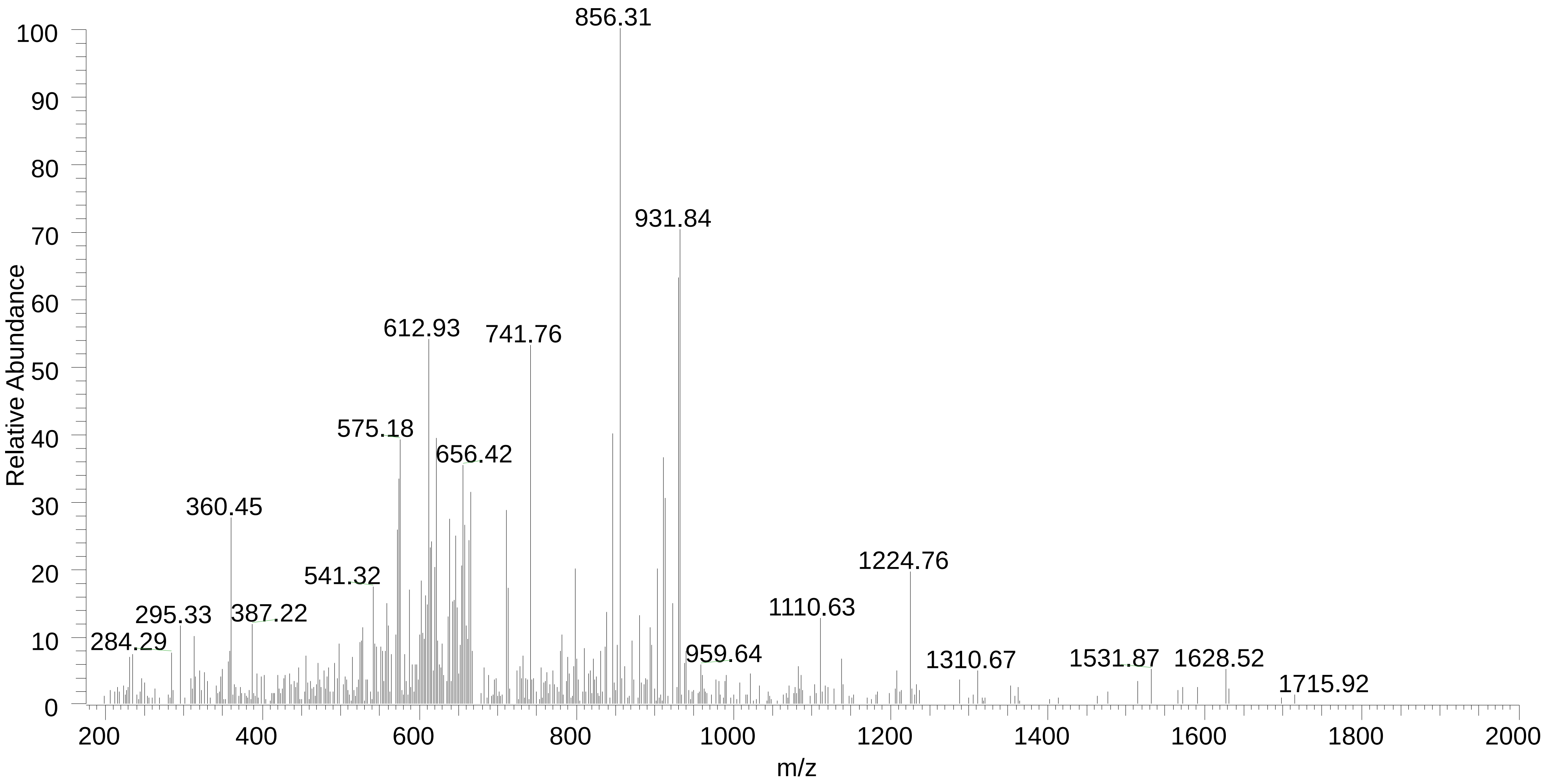

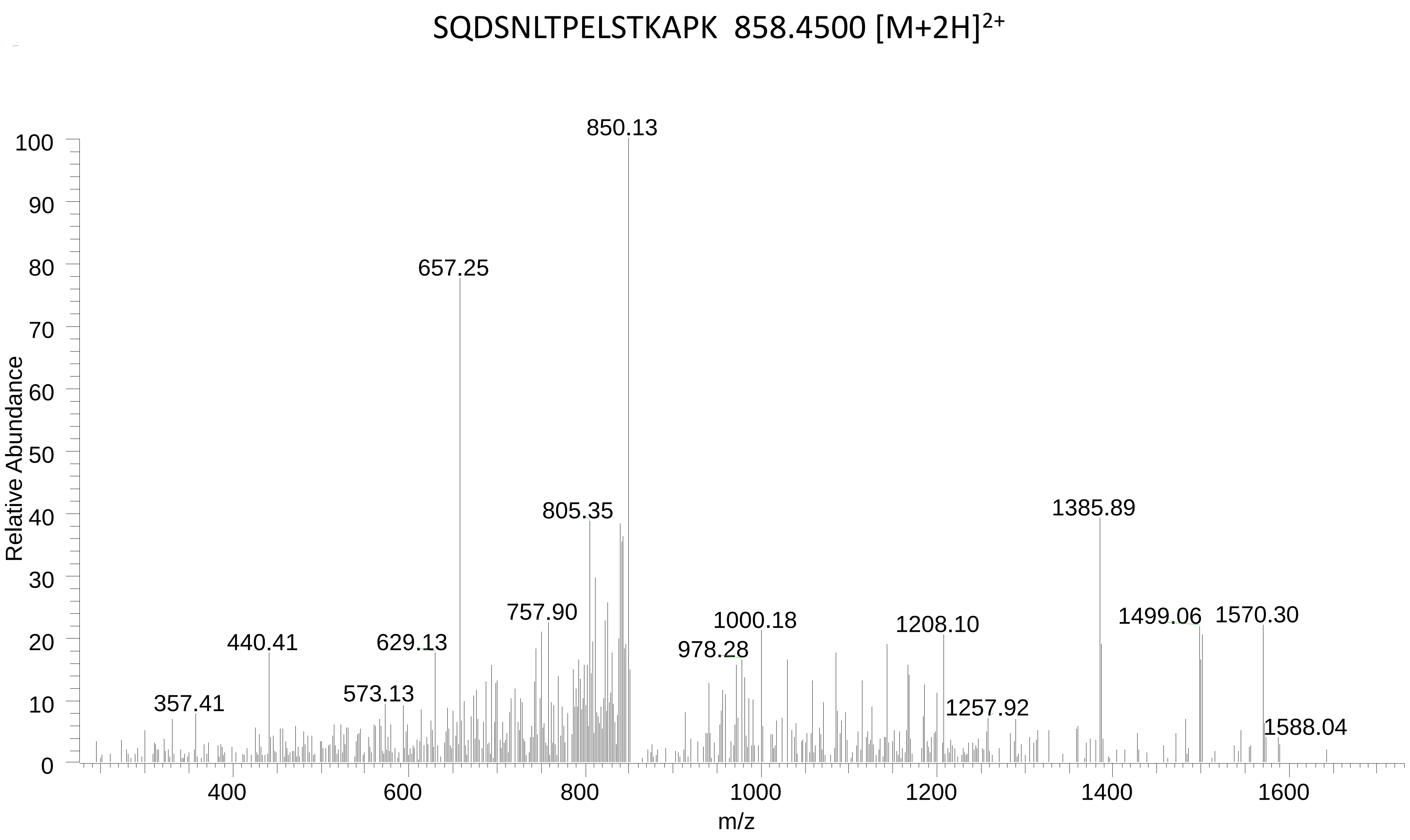
SQDSNLTPELSTKAPK 858.4500 [M+2H]2+
Relative Abundance
m/z
100
90
80
70
60
50
40
30
20
10
0
400
600
800
1000
1200
1400
1600
850.13
657.25
805.35
1385.89
757.90
1000.18
1208.10
1499.06
1570.30
440.41
629.13
978.28
357.41
573.13
1257.92
1588.04

ESINANTYINQNLEK 875.9343 [M+2H]$^{2+}$

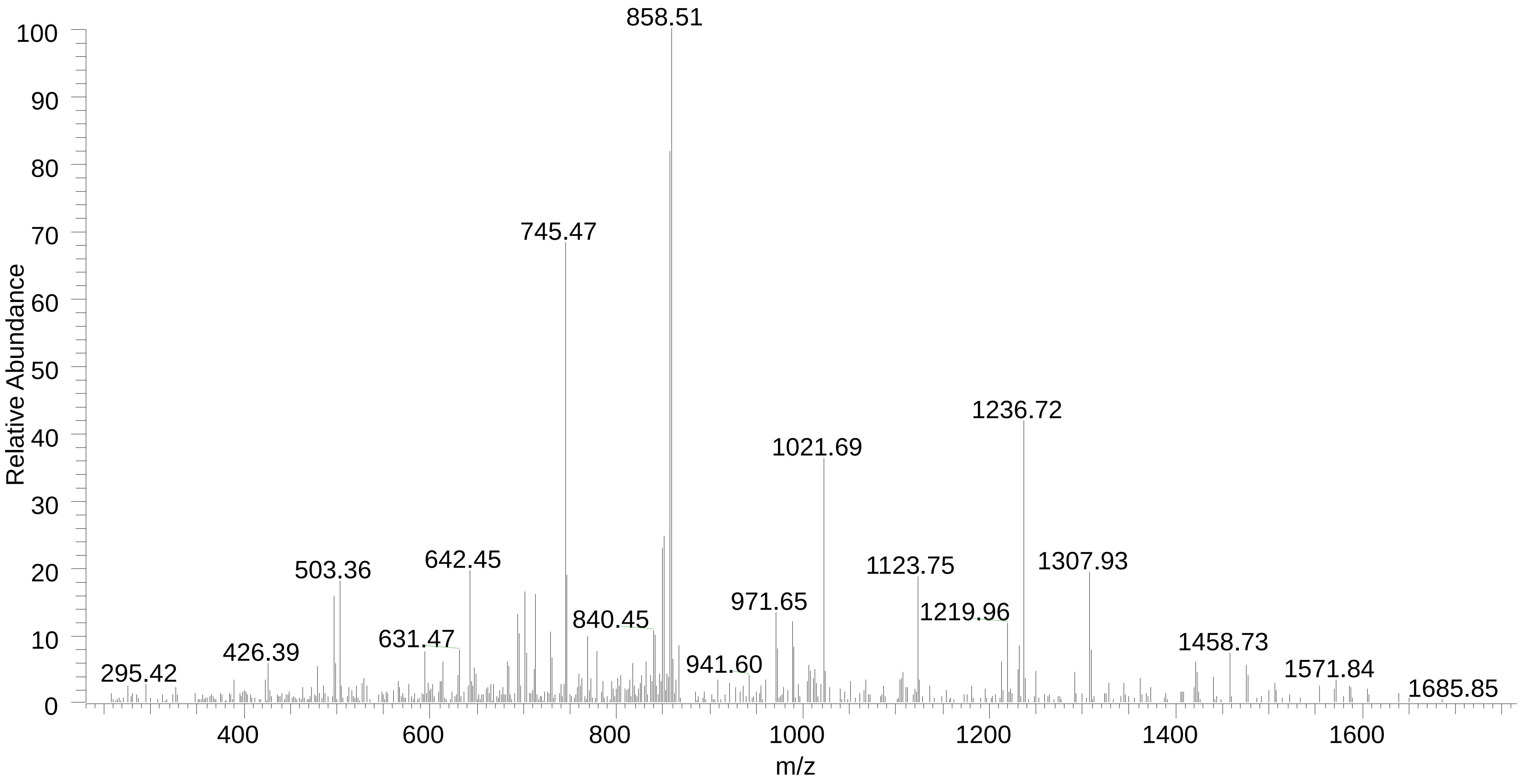

VAVLSTPLVTSFESK 789.4418 $[M+2H]^{2+}$

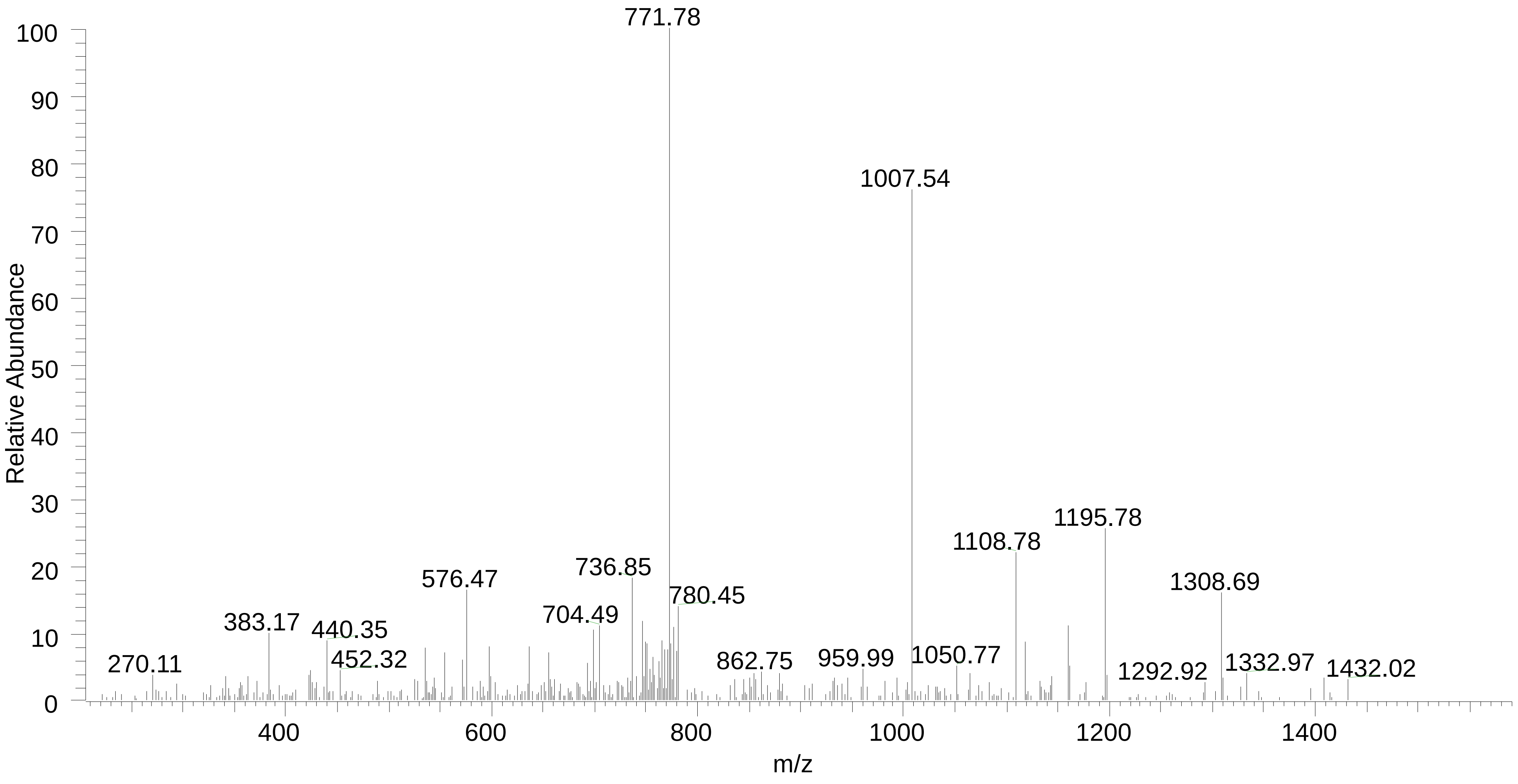

KDGEILFDAIDIYLRNK 675.0208 $[M+2H]^{2+}$

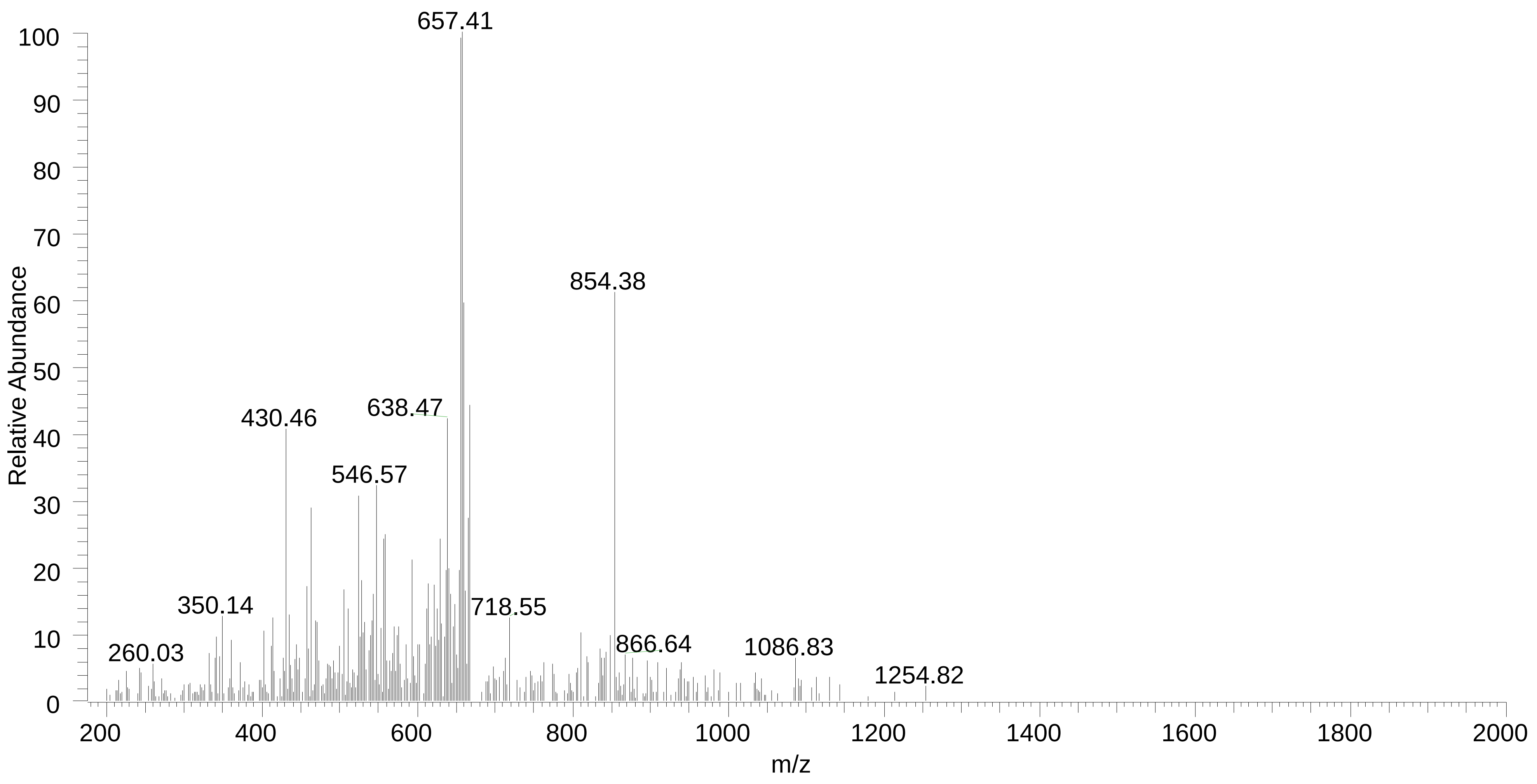

[42.01]MPVYKDGNTGKWYFSI   974.4655 $[M+2H]^{2+}$

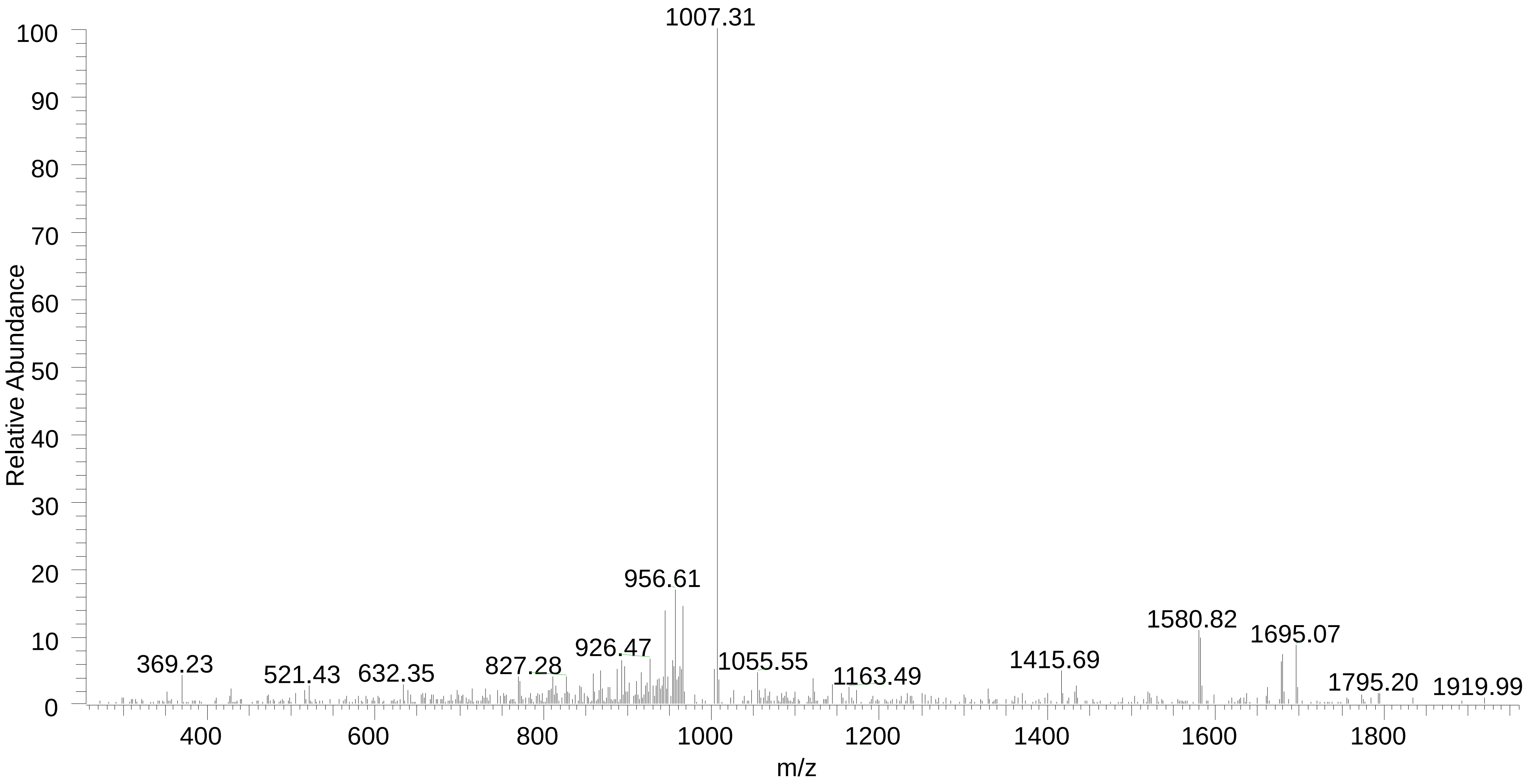

KTTSEALKEVLSDT 761.3987 $[M+2H]^{2+}$

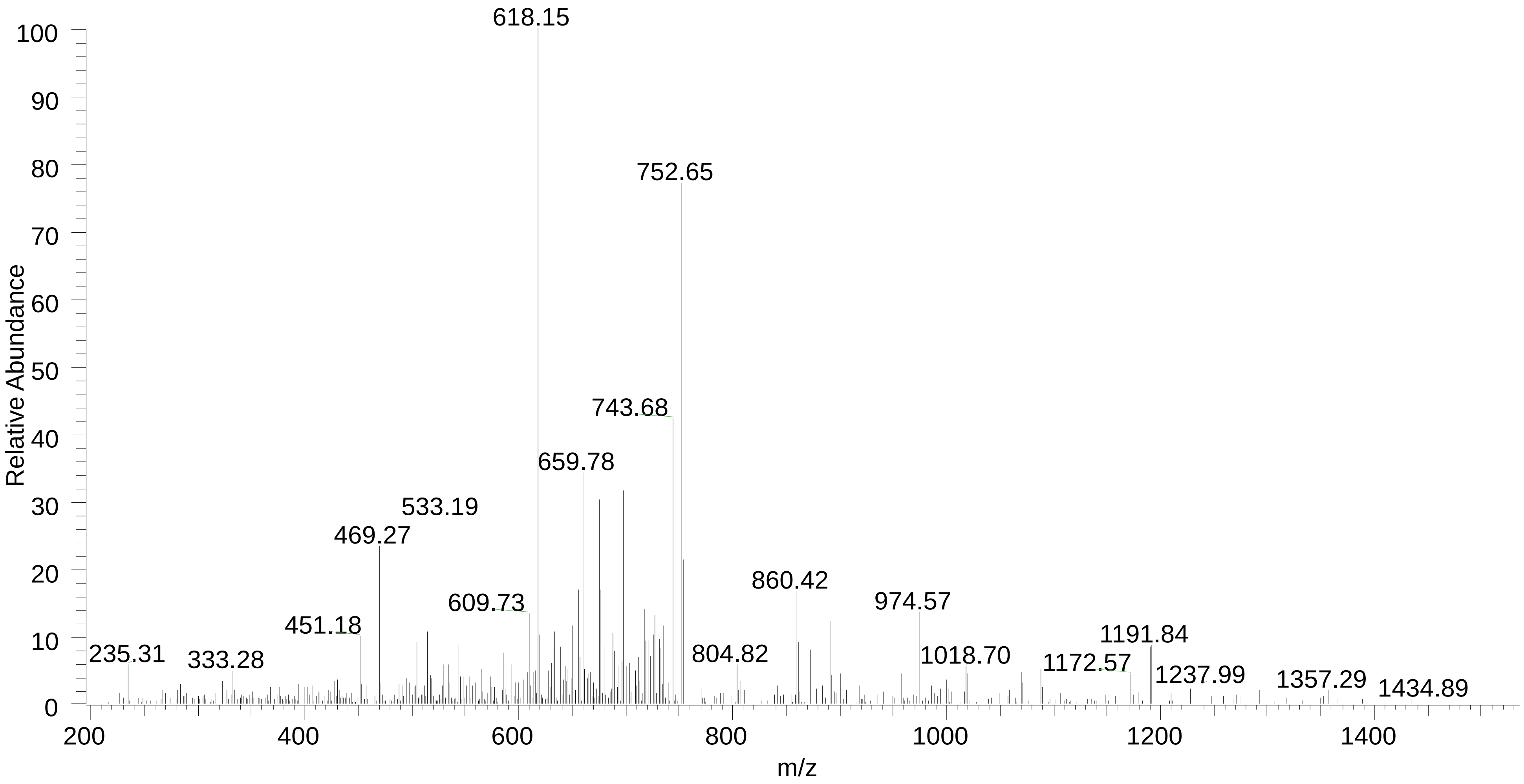

EPKPVDATGADDPLKPDDRMITNFHANLVDQKVSY 650.3128 $[M+3H]^{3+}$

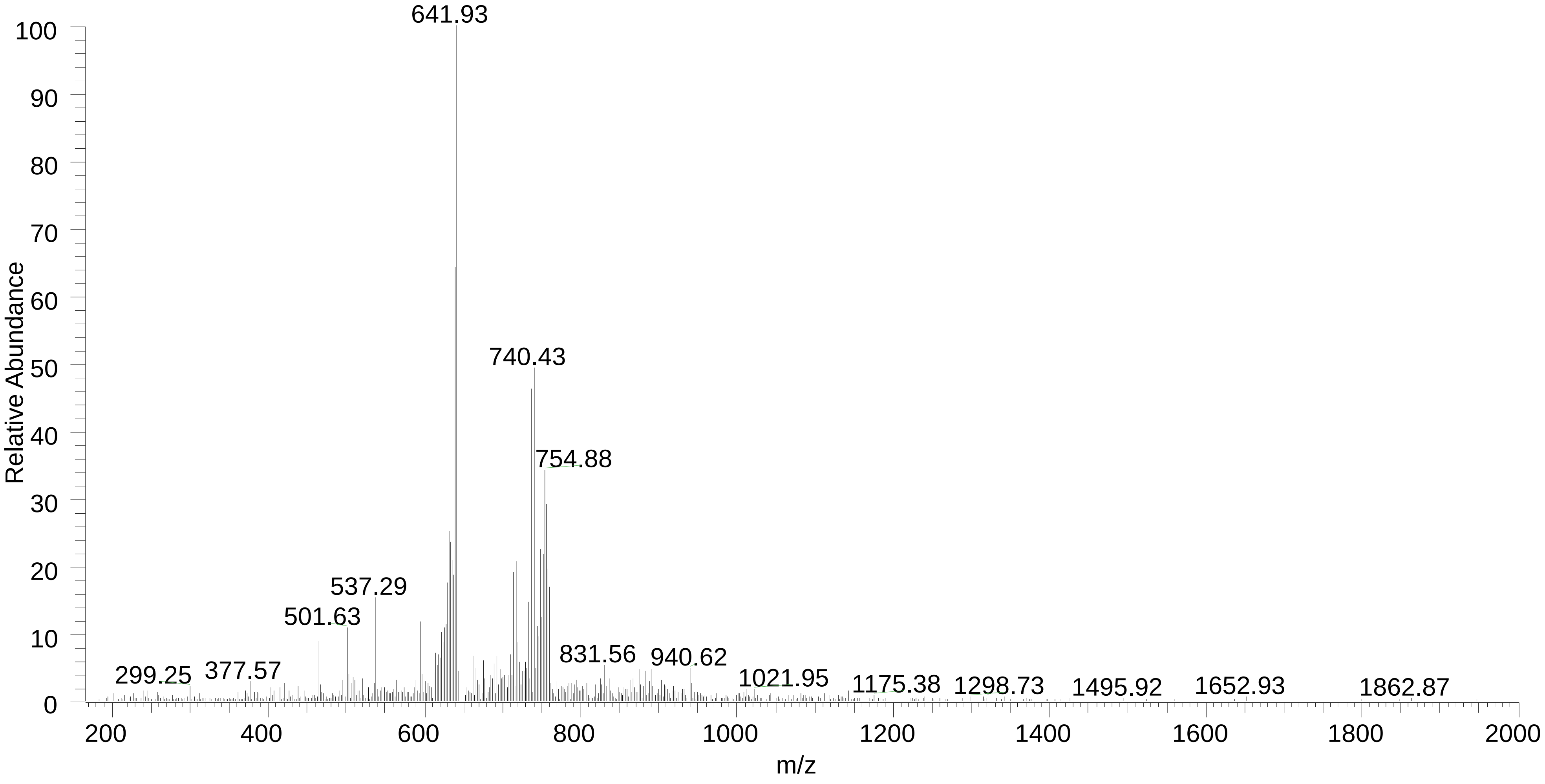

[42.01]MSHNALTTGIGIGAGAG 785.3987 $[M+2H]^{2+}$

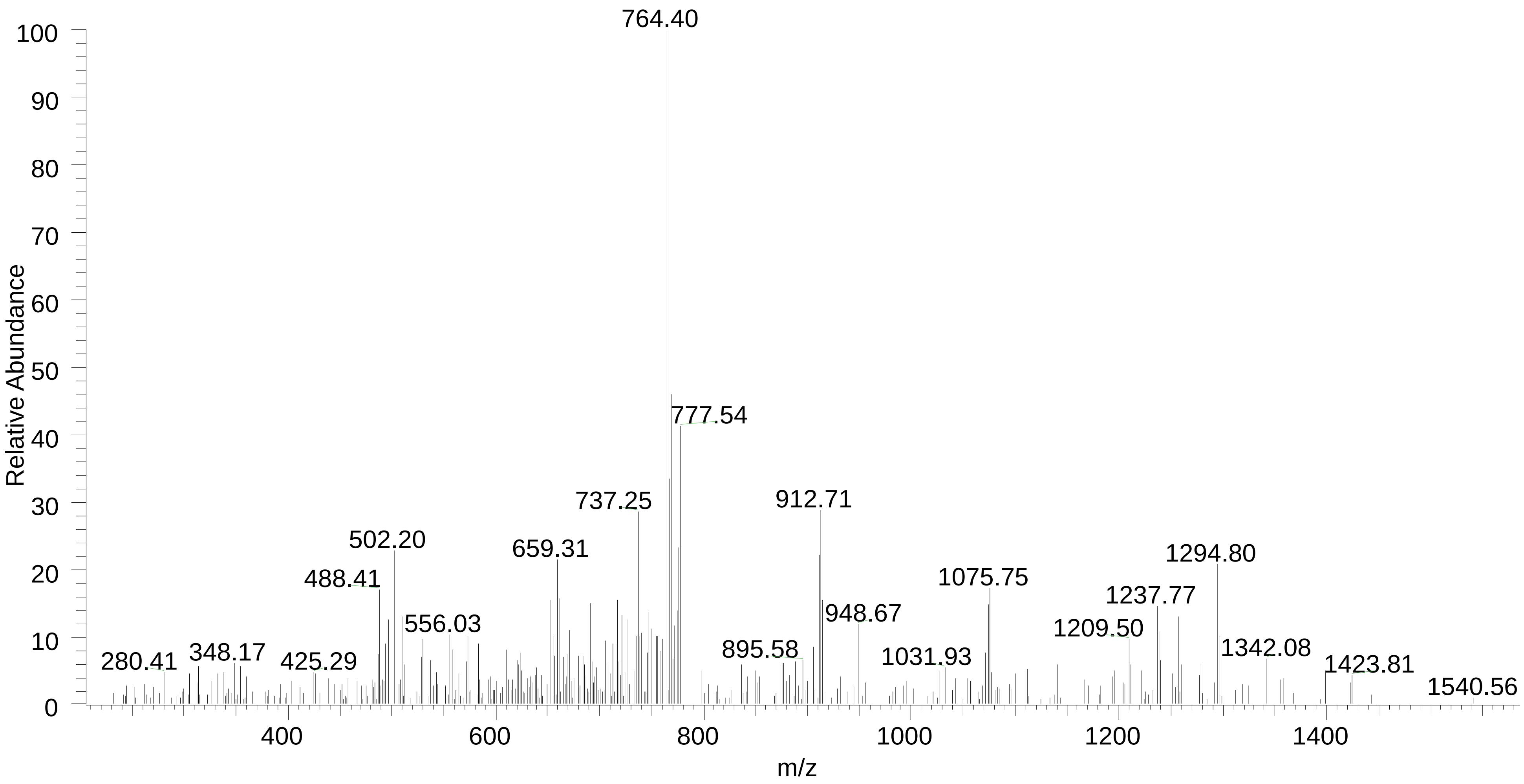

VQHPGKLVNKVM[147.04]SGLNINFGGGANATAK 946.8269 [M+2H]$^{2+}$

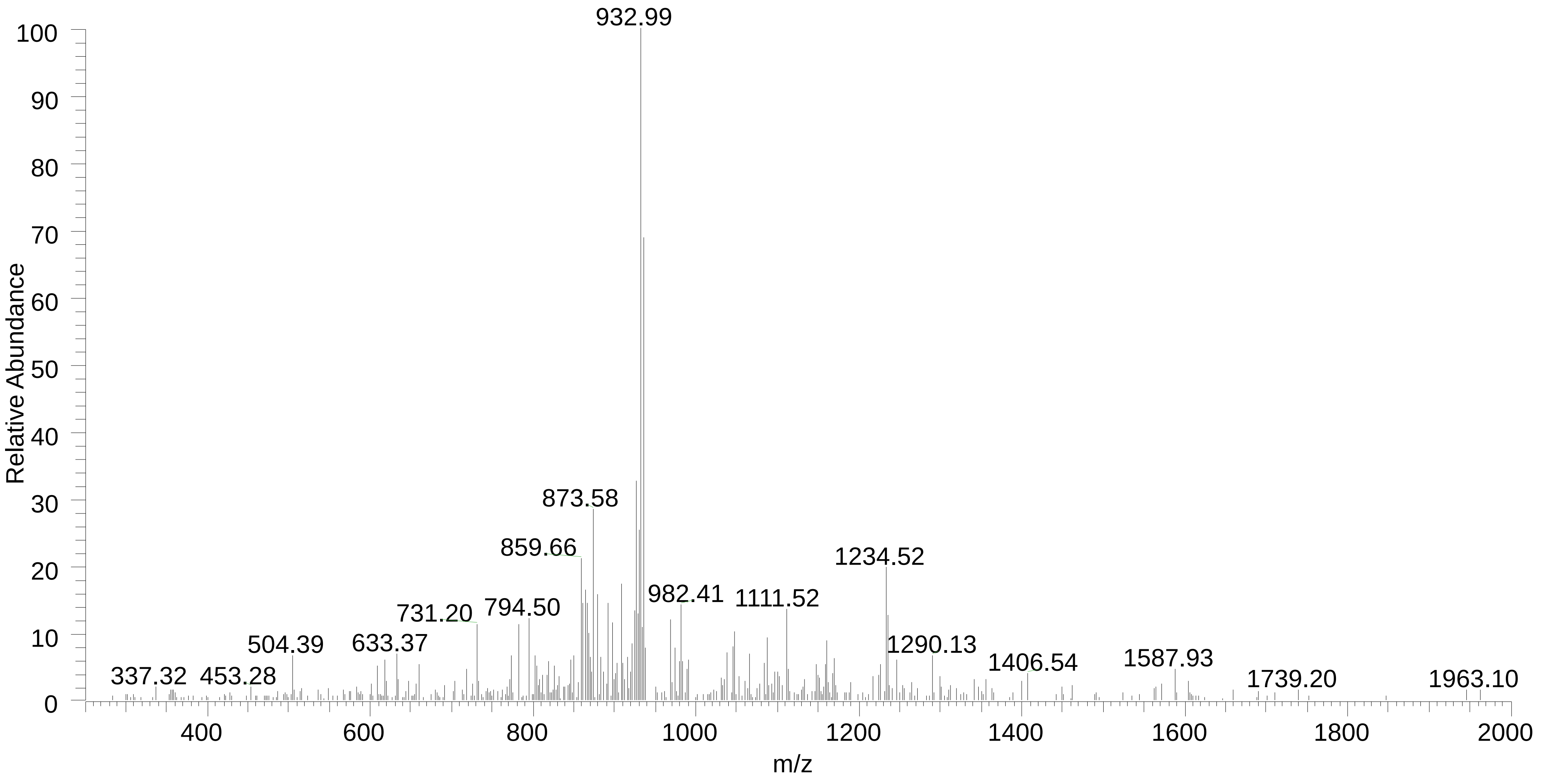

QM[147.04]MEGLSGVMDLAAVSGEDLGAVSDIVTDGLTAFGLKAKDSG 835.2061 $[M+2H]^{2+}$

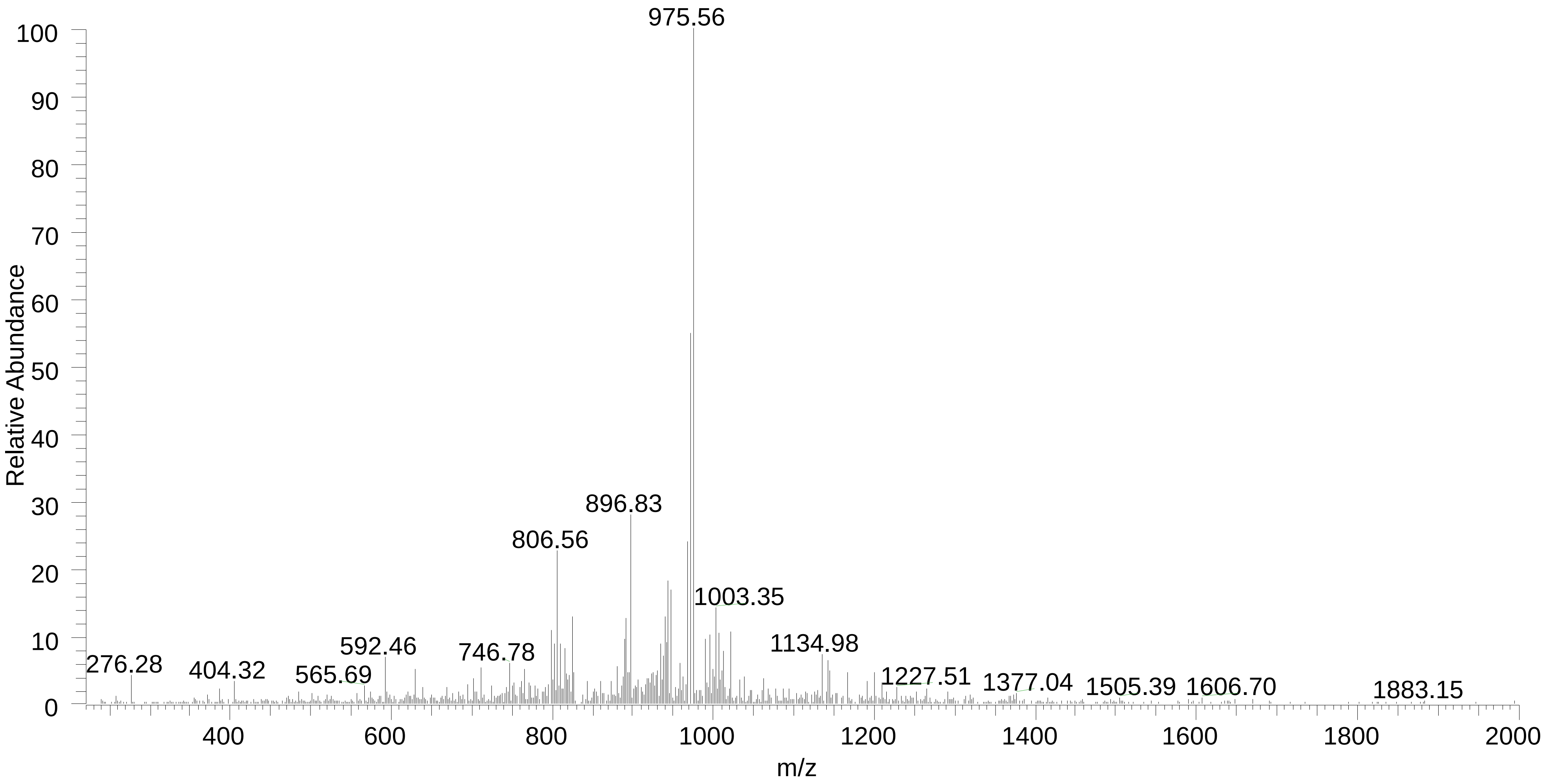

KSNVEAFSNAVK 647.3556 $[M+2H]^{2+}$

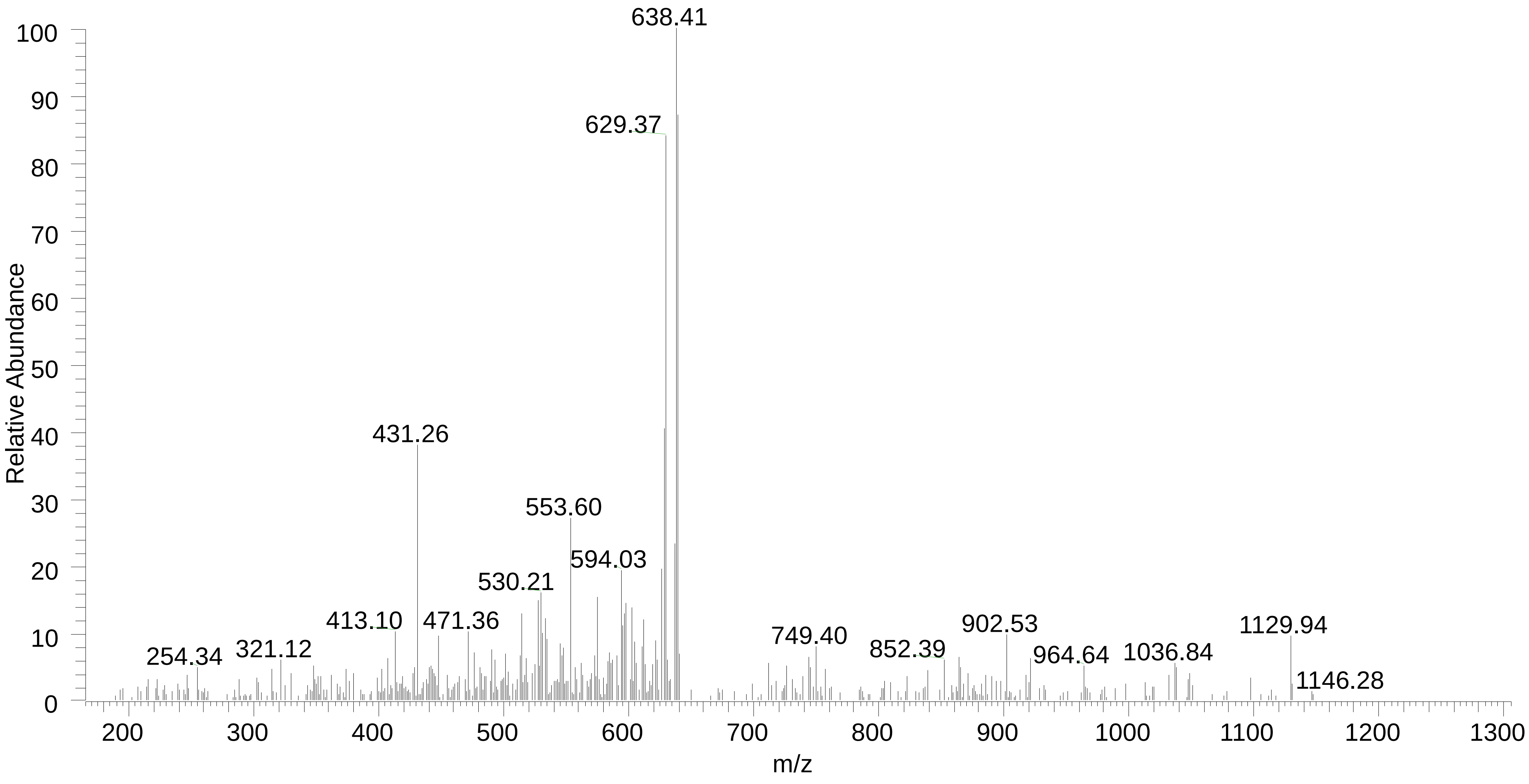

GMVASMQMQVVQVNVLTM[147.04]ELAQQNAMLTQQLTELK 785.1965 [M+2H]$^{2+}$

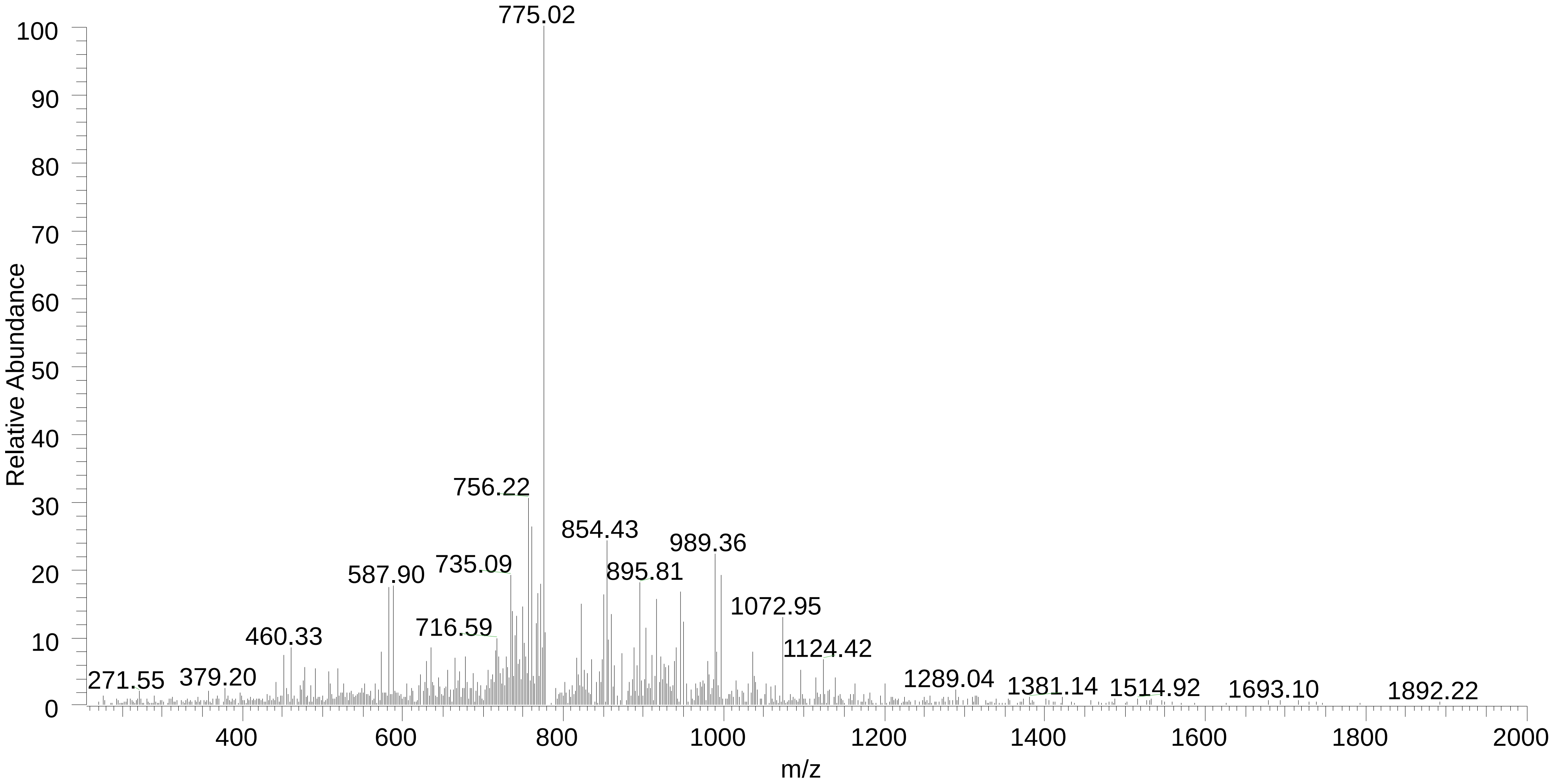

DIITVYC[160.03]PENGTATDEY 980.9245 [M+2H]$^{2+}$

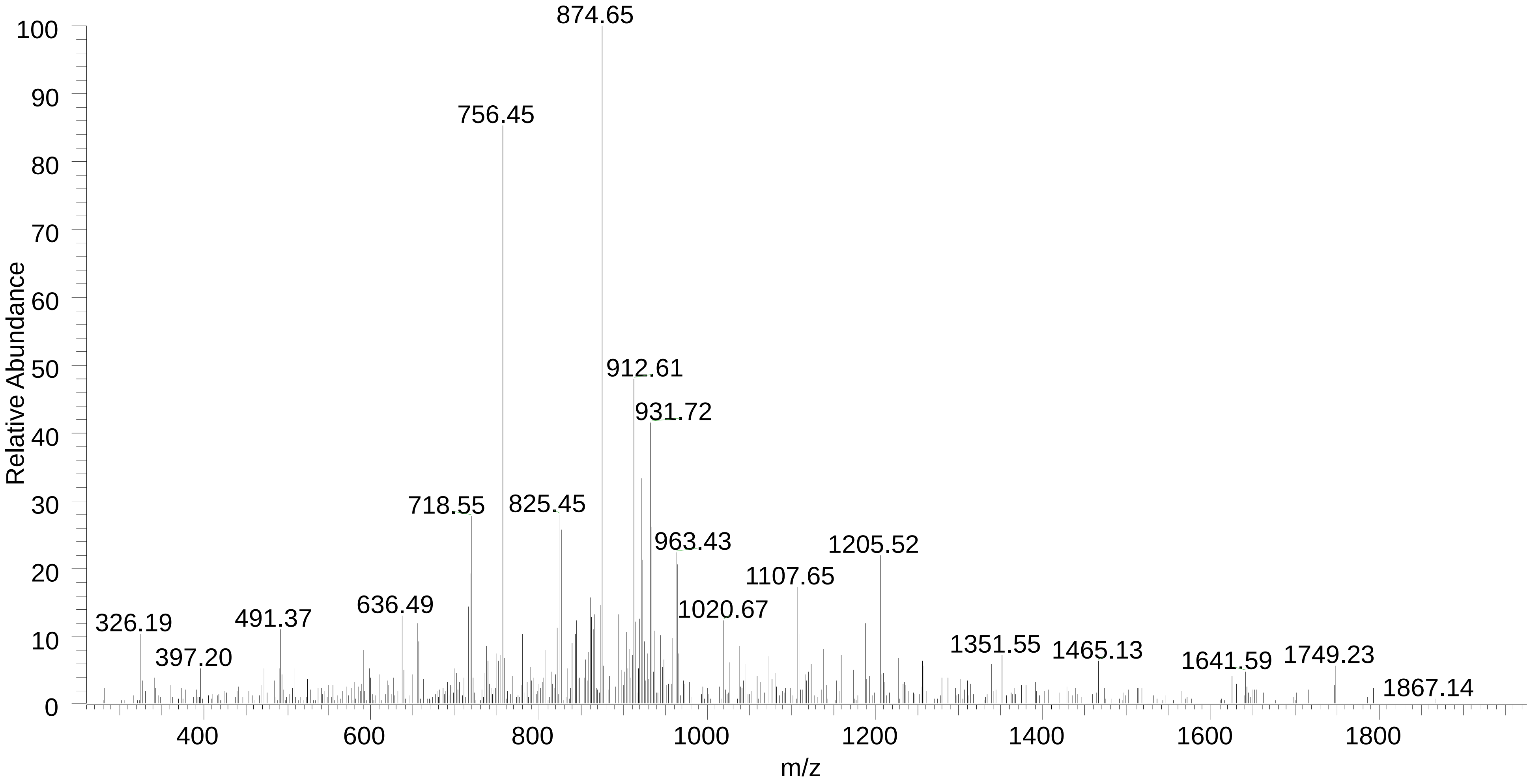

Supplement: Supplementary file 1 [file foods-10-00799-s001.zip › Figure S1_MC.pdf]
