# Supplementary material for: Proteomic Characterization of Bacteriophage Peptides from the Mastitis Producer Staphylococcus aureus by LC-ESI-MS/MS and the Bacteriophage Phylogenomic Analysis
Source: Foods. 2021 Apr 8;10(4):799. doi: 10.3390/foods10040799 (PMC8068337; doi:10.3390/foods10040799)
Supplement: Supplementary file 1 [file foods-10-00799-s001.zip › Table S1 and S2 in Supplemental Data 2.docx]

Supplementary Material

**Table S1.** *Staphylococcus aureus* (SA) strains used in this study. Total peptides were identified by LC-ESI-MS/MS and the number of phage peptides identified against UniProt/TrEMBL database from year 2020 which were analyzed by BLASTp. ATCC, American Type Culture Collection; CECT, Spanish Type Culture Collection.

| **Sample** | **Species** | **Strain** | **Source** | **NCBI accession number** |
| --- | --- | --- | --- | --- |
| **S1** | *Staphylococcus aureus* | SA_41 | Cheese/Goat |  |
| **S2** | *Staphylococcus aureus* | SA_92 | Cheese/Cow |  |
| **S3** | *Staphylococcus aureus* | SA_280 | Raw milk/Cow |  |
| **S4** | *Staphylococcus aureus* | SA_286 | Raw milk/Cow |  |
| **S5** | *Staphylococcus aureus* | SA_507 | Raw milk/Cow |  |
| **S6** | *Staphylococcus aureus* | SA_587 | Raw milk/Cow |  |
| **S7** | *Staphylococcus aureus* | SA_617 | Raw milk/Cow |  |
| **S8** | *Staphylococcus aureus* | SA_640 | Raw milk/Cow |  |
| **S9** | *Staphylococcus aureus* | SA_700 | Raw milk/Cow |  |
| **S10** | *Staphylococcus aureus* | SA_844 | Raw milk/Cow |  |
| **S11** | *Staphylococcus aureus* | SA_894 | Raw milk/Cow |  |
| **S12** | *Staphylococcus aureus* | SA_ATCC9144 | CECT 59/ATCC9144 | AY920400 |
| **S13** | *Staphylococcus aureus* | SA_ATCC29213 | CECT 794/ATCC29213 | LHUS00000000 |
| **S14** | *Staphylococcus aureus* | SA_ATCC35845 | CECT 4521/ATCC35845 |  |
| **S15** | *Staphylococcus aureus* | SA_CA19 | Cheese/Goat |  |
| **S16** | *Staphylococcus aureus* | SA_GP2 | Raw milk/Cow |  |
| **S17** | *Staphylococcus aureus* | SA_GP17 | Raw milk/Cow |  |
| **S18** | *Staphylococcus aureus* | SA_OV8 | Raw milk/Sheep |  |
| **S19** | *Staphylococcus aureus* | SA_PE1 | Raw milk/Sheep |  |
| **S20** | *Staphylococcus aureus* | SA_U17 | Human |  |

**Table S2.** Linage, authors and accession number of studied bacteriophages.

| **Phage name** | **Linage** | **Genome Accession number** | **References** |
| --- | --- | --- | --- |
| *Staphylococcus* virus 85 | dsDNA viruses, no RNA stage; *Caudovirales*; *Siphoviridae*; *Phietavirus* | NC_007050.1 | [55] |
| *Staphylococcus* phage phiSa2wa_st72 | dsDNA viruses, no RNA stage; *Caudovirales*; *Siphoviridae*; *Triavirus*; unclassified *Triavirus* | MG029513.1 | (O'Brien  et al., unpublished data) |
| *Staphylococcus* phage phiNM3 | dsDNA viruses, no RNA stage; *Caudovirales*; *Siphoviridae*; *Biseptimavirus*; unclassified *Biseptimavirus* | NC_008617. | [56] |
| *Staphylococcus* phage SH-St 15644 | dsDNA viruses, no RNA stage; *Caudovirales*; *Siphoviridae*; *Triavirus*; unclassified *Triavirus* | MG770897.1 | (Ji et al., unpublished data) |
| *Staphylococcus* phage phiN315 | dsDNA viruses, no RNA stage; *Caudovirales*; *Siphoviridae*; unclassified *Siphoviridae* | NC_004740.1 | [57] |
| *Staphylococcus* phage DW2 | dsDNA viruses, no RNA stage; *Caudovirales*; *Siphoviridae*; *Phietavirus*; unclassified Phietalikevirus | NC_024391.1 | [58] |
| *Staphylococcus* phage StauST398-2 | dsDNA viruses, no RNA stage; *Caudovirales*; *Siphoviridae*; *Triavirus*; unclassified *Triavirus* | NC_021323.1 | [59] |
| *Staphylococcus* virus 71 | dsDNA viruses, no RNA stage; *Caudovirales*; *Siphoviridae*; *Phietavirus* | NC_007059.1 | [56] |
| *Staphylococcus* virus IPLA35 | dsDNA viruses, no RNA stage; *Caudovirales*; *Siphoviridae*; *Triavirus* | NC_011612.1 | [60] |
| *Staphylococcus* phage SMSAP5 | dsDNA viruses, no RNA stage; *Caudovirales*; *Siphoviridae*; *Triavirus*; unclassified *Triavirus* | NC_019513.1 | (Lee et al., unpublished data) |
| *Staphylococcus* virus phiETA2 | dsDNA viruses, no RNA stage; *Caudovirales*; *Siphoviridae*; *Phietavirus* | NC_008798.1 | (Sawada et al., unpublished data) |
| *Staphylococcus* phage phiSa2wa_st121mssa | dsDNA viruses, no RNA stage; *Caudovirales*; *Siphoviridae*; *Triavirus*; unclassified *Triavirus* | MG029518.1 | (O'Brien et al., unpublished data) |
| *Staphylococcus* phage vB_SauS_phi2 | dsDNA viruses, no RNA stage; *Caudovirales*; *Siphoviridae*; *Triavirus*; unclassified *Triavirus* | NC_028862.1 | (El Haddad et al., unpublished data) |
| *Staphylococcus* virus 88 | dsDNA viruses, no RNA stage; *Caudovirales*; *Siphoviridae*; *Phietavirus* | NC_007063.1 | [55] |
| *Staphylococcus* phage SP5 | dsDNA viruses, no RNA stage; *Caudovirales*; *Siphoviridae*; *Phietavirus*; unclassified Phietalikevirus | JX274646.1 | [61] |
| *Staphylococcus* phage tp310-2 | dsDNA viruses, no RNA stage; *Caudovirales*; *Siphoviridae*; *Triavirus*; unclassified *Triavirus* | NC_009762.3 | (Reichholf et al., unpublished data) |
| *Staphylococcus* phage StauST398-4 | dsDNA viruses, no RNA stage; *Caudovirales*; *Siphoviridae*; *Biseptimavirus*; unclassified *Biseptimavirus* | NC_023499.1 | [59] |
| *Staphylococcus* virus phi12 | dsDNA viruses, no RNA stage; *Caudovirales*; *Siphoviridae*; *Triavirus* | NC_004616.1 | [62] |
| *Staphylococcus* virus IPLA88 | dsDNA viruses, no RNA stage; *Caudovirales*; *Siphoviridae*; *Phietavirus* | NC_011614.1 | [60] |
| *Staphylococcus* virus 55 | dsDNA viruses, no RNA stage; *Caudovirales*; *Siphoviridae*; *Phietavirus* | NC_007060.1 | [55] |
| *Staphylococcus* phage phiNM | dsDNA viruses, no RNA stage; *Caudovirales*; *Siphoviridae*; unclassified *Siphoviridae* |  |  |
| *Staphylococcus* phage phi7247PVL | dsDNA viruses, no RNA stage; *Caudovirales*; *Siphoviridae*; *Biseptimavirus*; unclassified *Biseptimavirus* | AP011956.1 | [63] |
| *Staphylococcus* virus 3a | dsDNA viruses, no RNA stage; *Caudovirales*; *Siphoviridae*; *Triavirus* | NC_007053.1 | [55] |
| *Staphylococcus* virus SAP26. | dsDNA viruses, no RNA stage; *Caudovirales*; *Siphoviridae*; *Phietavirus* | NC_014460.1 | (Rahman et al.,unpublished data) |
| *Staphylococcus* phage LH1 | dsDNA viruses, no RNA stage; *Caudovirales*; *Siphoviridae*; *Triavirus*; unclassified *Triavirus* | JX174275.1 | [64] |
| *Staphylococcus* phage phiSa2wa_st22 | dsDNA viruses, no RNA stage; *Caudovirales*; *Siphoviridae*; *Biseptimavirus*; unclassified *Biseptimavirus* | MG029510.1 | (O'Brien et al., unpublished data) |
| *Staphylococcus* virus 77 | dsDNA viruses, no RNA stage; *Caudovirales*; *Siphoviridae*; *Biseptimavirus* | NC_005356.1 | [65] |
| *Staphylococcus* phage P240 | sDNA viruses, no RNA stage; *Caudovirales*; *Siphoviridae*; *Triavirus*; unclassified *Triavirus* | KY056620.1 | [66] |
| *Staphylococcus* virus phiSLT | dsDNA viruses, no RNA stage; *Caudovirales*; *Siphoviridae*; *Triavirus* | NC_002661.2 | [67] |
| *Staphylococcus* phage phiSa2wa_st30 | dsDNA viruses, no RNA stage; *Caudovirales*; *Siphoviridae*; *Triavirus*; unclassified *Triavirus* | MG029511.1 | (O'Brien et al., unpublished data) |
| *Staphylococcus* phage P954 | dsDNA viruses, no RNA stage; *Caudovirales*; *Siphoviridae*; *Biseptimavirus*; unclassified *Biseptimavirus* | NC_013195.1 | (Young and Sum­mers unpublished data) |
| *Staphylococcus* phage R4 | Viruses; unclassified viruses; unclassified bacterial viruses. | MT366568.1 | (Petrics et al., unpublished data) |
| *Staphylococcus* phage vB_SauS_JS02 | dsDNA viruses, no RNA stage; *Caudovirales*; *Siphoviridae*; *Triavirus*; unclassified *Triavirus* | BCD56354.1 | (Zhang et al., unpublished data) |
| *Staphylococcus* phage SA137ruMSSAST121PVL | dsDNA viruses, no RNA stage; *Caudovirales*; *Siphoviridae*; *Triavirus*; unclassified *Triavirus* | MH384261.1 | (Dmitrenko et al., unpublished data) |
| *Staphylococcus* phage vB_SauS_fPfSau02 | dsDNA viruses, no RNA stage; *Caudovirales*; *Siphoviridae*; *Triavirus*; unclassified *Triavirus* | MK348510.1 | (Kiljunen et al., unpublished data) |
| *Staphylococcus* phage P282 | dsDNA viruses, no RNA stage; *Caudovirales*; *Siphoviridae*; *Biseptimavirus*; unclassified *Biseptimavirus* | KT809368.1 | [66] |
| *Staphylococcus* virus Baq_Sau1 | dsDNA viruses, no RNA stage; *Caudovirales*; *Siphoviridae*; *Phietavirus* | MK658834.1 | (Lozano-Solano et al., unpublished data) |
| *Staphylococcus* phage SA12 | dsDNA viruses, no RNA stage; *Caudovirales*; *Siphoviridae*; *Phietavirus* | KC677663.1 | [68] |
| *Staphylococcus* phage phiSa2wa_st1 | dsDNA viruses, no RNA stage; *Caudovirales*; *Siphoviridae*; *Triavirus*; unclassified *Triavirus* | MF580410.1 | (O'Brien et al., unpublished data) |
| *Staphylococcus phage* Henu2 | dsDNA viruses, no RNA stage; *Caudovirales*; *Siphoviridae*; *Phietavirus* | MK211557.1 | (Liu and Teng unpublished data) |
| *Staphylococcus* phage tp310-1 | dsDNA viruses, no RNA stage; *Caudovirales*; *Siphoviridae*; *Biseptimavirus*; unclassified *Biseptimavirus* | NC_009761.3 | (Reichholf,U et al., unpublished data) |
| *Staphylococcus* phage StauST398-1 | dsDNA viruses, no RNA stage; *Caudovirales*; *Siphoviridae*; *Phietavirus* | NC_021326.1 | [59] |
| *Staphylococcus* phage phi2958PVL | dsDNA viruses, no RNA stage; *Caudovirales*; *Siphoviridae*; *Triavirus*; unclassified *Triavirus* | NC_011344.1 | [69] |
| *Staphylococcus* phage PVL | dsDNA viruses, no RNA stage; *Caudovirales*; *Siphoviridae*; *Biseptimavirus*; unclassified *Biseptimavirus* | NC_002321.1 | [70] |
| *Staphylococcus* virus 13 | dsDNA viruses, no RNA stage; *Caudovirales*; *Siphoviridae*; *Biseptimavirus*; unclassified *Biseptimavirus* | NC_004617.1 | [62] |
| *Staphylococcus* phage ROSA | dsDNA viruses, no RNA stage; *Caudovirales*; *Siphoviridae*; *Phietavirus* | NC_007058.1 | [55] |
| *Staphylococcus* phage phi7401PVL | dsDNA viruses, no RNA stage; *Caudovirales*; *Siphoviridae*; *Triavirus*; unclassified *Triavirus* | NC_020199.1 | [71] |
| *Staphylococcus* virus 80 | dsDNA viruses, no RNA stage; *Caudovirales*; *Siphoviridae*; *Phietavirus* | NC_030652.1 | [72] |
| Staphylococcus phage phiETA3 | dsDNA viruses, no RNA stage; *Caudovirales*; *Siphoviridae*; *Phietavirus* | NC_008799.1 | (Sawada, unpublished data) |
| Staphylococcus phage 55-2 | dsDNA viruses, no RNA stage; *Caudovirales*; *Siphoviridae*; *Phietavirus* | KR709302.1 | [73] |
| *Staphylococcus phage* B166 | dsDNA viruses, no RNA stage; *Caudovirales*; *Siphoviridae*; *Phietavirus* | NC_028859.1 | [74] |
| Staphylococcus phage B236 | dsDNA viruses, no RNA stage; *Caudovirales*; *Siphoviridae*; *Phietavirus* | NC_028915.1 | [74] |
| Staphylococcus phage 11 | dsDNA viruses, no RNA stage; *Caudovirales*; *Siphoviridae*; *Phietavirus* | NC_004615.1 | [62] |
| Staphylococcus phage phiETA | dsDNA viruses, no RNA stage; *Caudovirales*; *Siphoviridae*; *Phietavirus* | NC_003288.1 | [75] |
| Staphylococcus phage 69 | dsDNA viruses, no RNA stage; *Caudovirales*; *Siphoviridae*; *Phietavirus* | NC_007048.1 | [55] |
| Staphylococcus phage TEM123 | dsDNA viruses, no RNA stage; *Caudovirales*; *Siphoviridae*; *Phietavirus* | NC_017968.1 | (Lee and Park unpublished data) |
| Staphylococcus phage 92 | dsDNA viruses, no RNA stage; *Caudovirales*; *Siphoviridae*; *Phietavirus* | NC_007064.1 | [55] |
| Staphylococcus phage phiNM2 | dsDNA viruses, no RNA stage; *Caudovirales*; *Siphoviridae*; *Phietavirus* | NC_028913.1 | [56] |
| Staphylococcus phage phiNM1 | dsDNA viruses, no RNA stage; *Caudovirales*; *Siphoviridae*; *Phietavirus* | NC_008583.1 | [56] |
| Staphylococcus phage 29 | dsDNA viruses, no RNA stage; *Caudovirales*; *Siphoviridae*; *Phietavirus* | NC_007061.1 | [55] |
| Staphylococcus phage vB_SauS-SAP27 | dsDNA viruses, no RNA stage; *Caudovirales*; *Siphoviridae*; *Phietavirus* | MN904510.1 | (Park and Park unpublished data) |
| Staphylococcus virus 80alpha | dsDNA viruses, no RNA stage; *Caudovirales*; *Siphoviridae*; *Phietavirus* | NC_009526.1 | [72] |
| Staphylococcus phage SAP33 | dsDNA viruses, no RNA stage; *Caudovirales*; *Siphoviridae*; *Phietavirus* | MK801682.1 | (Yu unpublished data) |
| Staphylococcus phage 3MRA | dsDNA viruses, no RNA stage; *Caudovirales*; *Siphoviridae*; *Phietavirus* | NC_028917.1 | [76] |
| Staphylococcus virus phiMR11 | dsDNA viruses, no RNA stage; *Caudovirales*; *Siphoviridae*; *Phietavirus* | NC_010147.1 | [77] |
| Staphylococcus phage HSA84 | dsDNA viruses, no RNA stage; *Caudovirales*; *Siphoviridae*; *Phietavirus* | MG557619.1 | (Seo et al., unpublished data) |
| *Staphylococcus* phage phi-42 | Viruses; unclassified viruses; unclassified bacterial viruses. | U01872.1 | [78] |
| Staphylococcus phage phiSa2wa_st80 | dsDNA viruses, no RNA stage; *Caudovirales*; *Siphoviridae*; *Triavirus*; unclassified *Triavirus* | MG029515.1 | (O'Brien unpublished data) |
| *Staphylococcus* virus 69 | dsDNA viruses, no RNA stage; *Caudovirales*; *Siphoviridae*; *Phietavirus* | NC_007048.1 | [55] |
| *Staphylococcus* virus phiMR25 | dsDNA viruses, no RNA stage; *Caudovirales*; *Siphoviridae*; *Phietavirus* | NC_010808.1 | [79] |
| Staphylococcus phage phiJB | dsDNA viruses, no RNA stage; *Caudovirales*; *Siphoviridae*; *Phietavirus* | NC_028669.1 | [80] |
| *Staphylococcus* phage SA45ruMSSAST97 | dsDNA viruses, no RNA stage; Caudovirales; Siphoviridae; Biseptimavirus; unclassified Biseptimavirus |  | (Dmitrenko et al., unpublished data) |
| *Staphylococcus* virus 47 | dsDNA viruses, no RNA stage; *Caudovirales*; *Siphoviridae*; *Triavirus*; unclassified *Triavirus* | NC_007054.1 | [55] |
| *Staphylococcus* phage SAP40 | dsDNA viruses, no RNA stage; *Caudovirales*; *Siphoviridae*; *Phietavirus* | MK801683.1 | (Yu, umplublished data) |
| *Staphylococcus* phage phi 53 | dsDNA viruses, no RNA stage; *Caudovirales*; *Siphoviridae* |  | [81] |
| *Staphylococcus* phage SPbeta-like | dsDNA viruses, no RNA stage; *Caudovirales*; *Siphoviridae;* Spbetavirus; unclassified pbetalikevirus. | NC_029119.1 | (Kornyenko  et al., unpublished data) |
| *Staphylococcus* phage phiSa2wa_st5 | dsDNA viruses, no RNA stage; *Caudovirales*; *Siphoviridae*; *Triavirus*; unclassified *Triavirus* | MG029509.1 | (O'Brien unpublished data) |
| *Staphylococcus phage phi3A* | dsDNA viruses, no RNA stage; *Caudovirales*; *Siphoviridae* | AAM49603.1 | [81] |
| *Staphylococcus phage SA97* | dsDNA viruses, no RNA stage; *Caudovirales*; *Siphoviridae*; *Phietavirus* | NC_029010.1 | [82] |
| *Staphylococcus virus 53* | dsDNA viruses, no RNA stage; *Caudovirales*; *Siphoviridae*; *Phietavirus* | NC_007049.1 | [55] |
| *Staphylococcus phage SA75* | dsDNA viruses, no RNA stage; *Caudovirales*; *Siphoviridae*; *Phietavirus* | MT013111.1 | (DSouza,unpublished data) |
| *Staphylococcus phage SA13* | dsDNA viruses, no RNA stage; *Caudovirales*; *Siphoviridae*; *Phietavirus* | NC_021863.1 | (Shin, unpublished data) |
| *Staphylococcus phage P630* | dsDNA viruses, no RNA stage; Caudovirales; Siphoviridae; Biseptimavirus; unclassified Biseptimavirus | NC_048635.1 | [66] |
| *Staphylococcus phage IME1361_01* | dsDNA viruses, no RNA stage; Caudovirales; Siphoviridae; Biseptimavirus; unclassified Biseptimavirus | NC_048657.1 | (Sun, unpublished data) |
| *Staphylococcus phage SAP090B* | dsDNA viruses, no RNA stage; Caudovirales; Siphoviridae; Biseptimavirus; unclassified Biseptimavirus | GQ915271.1 | (Summers et al., unpublished data) |
| *Staphylococcus phage IME1346_01* | dsDNA viruses, no RNA stage; Caudovirales; Siphoviridae; Biseptimavirus; unclassified Biseptimavirus | KY653125.1 | (Sun et al, unpublished data) |
| *Staphylococcus phage P1105* | dsDNA viruses, no RNA stage; Caudovirales; Siphoviridae; Biseptimavirus; unclassified Biseptimavirus | NC_048636.1 | [66] |
| *Staphylococcus phage phi5967PVL* | dsDNA viruses, no RNA stage; Caudovirales; Siphoviridae; Biseptimavirus; unclassified Biseptimavirus | NC_019921.1 | [63] |
| *Staphylococcus* virus 108PVL | dsDNA viruses, no RNA stage; Caudovirales; Siphoviridae; Biseptimavirus; unclassified Biseptimavirus | NC_008689.1 | [69] |
| *Staphylococcus* phage 3 AJ-2017 | dsDNA viruses, no RNA stage; Caudovirales; Siphoviridae; Biseptimavirus; unclassified Biseptimavirus | NC_048644.1 | (Escobar-Perez un published data) |
| *Staphylococcus* prophage phiPV83 | dsDNA viruses, no RNA stage; Caudovirales; Siphoviridae; Biseptimavirus; unclassified Biseptimavirus | NC_002486.1 | [83] |
| *Staphylococcus* phage phiBU01 | dsDNA viruses, no RNA stage; Caudovirales; Siphoviridae; Biseptimavirus; unclassified Biseptimavirus | NC_026016.1 | [84] |
| *Staphylococcus* phage phiSa119 | dsDNA viruses, no RNA stage; Caudovirales; Siphoviridae; Biseptimavirus; unclassified Biseptimavirus | NC_025460.1 | [85] |
| *Staphylococcus* phage 23MRA | dsDNA viruses, no RNA stage; Caudovirales; Siphoviridae; Biseptimavirus; unclassified Biseptimavirus | NC_028775.1 | [76] |
| *Staphylococcus* phage SA7 | dsDNA viruses, no RNA stage; Caudovirales; Siphoviridae; Biseptimavirus; unclassified Biseptimavirus | NC_048658.1 | (Kim, unpublished data) |
| *Staphylococcus* phage JS01 | dsDNA viruses, no RNA stage; Caudovirales; Siphoviridae; Biseptimavirus; unclassified Biseptimavirus | NC_021773.2 | [86] |
| *Staphylococcus* phage SA345ruMSSAST8 | dsDNA viruses, no RNA stage; Caudovirales; Siphoviridae; Biseptimavirus; unclassified Biseptimavirus | NC_048713.1 | (Dmitrenko et al., un published data) |
| *Staphylococcus phage SA1014ruMSSAST7* | dsDNA viruses, no RNA stage; Caudovirales; Siphoviridae; Biseptimavirus; unclassified Biseptimavirus | NC_048710.1 | (Dmitrenko et al., un published data) |
| *Staphylococcus phage SA780ruMSSAST101* | dsDNA viruses, no RNA stage; Caudovirales; Siphoviridae; Biseptimavirus; unclassified Biseptimavirus | NC_048711.1 | (Dmitrenko et al., unpublished data) |
| *Staphylococcus phage YMC/09/04/R1988* | dsDNA viruses, no RNA stage; *Caudovirales*; *Siphoviridae*; *Triavirus*; unclassified *Triavirus* | NC_022758.1 | [87] |
| *Staphylococcus phage phi879* | dsDNA viruses, no RNA stage; Caudovirales; Siphoviridae; Biseptimavirus; unclassified Biseptimavirus | KY389064.1 | [88] |
| *Staphylococcus phage phi575* | dsDNA viruses, no RNA stage; Caudovirales; Siphoviridae; Biseptimavirus; unclassified Biseptimavirus | KY389063.1 | [88] |
